# Glycan atlassing enables functional tracing of cell state

**DOI:** 10.1038/s41565-026-02151-y

**Published:** 2026-05-14

**Authors:** Dijo Moonnukandathil Joseph, Nazlican Yurekli, Sarah Fritsche, Reem Hashem, Oana-Maria Thoma, Imen Larafa, Tina Boric, Chloé Bielawski, Karim Almahayni, Kristian Franze, Maximilian J. Waldner, Leonhard Möckl

**Affiliations:** 1 Max Planck Institute for the Science of Light, Erlangen, Germany; 2 Faculty of Medicine/CITABLE, University Hospital Erlangen, FAU Erlangen-Nuremberg, Erlangen, Germany; 3 Faculty of Sciences/Department of Physics, FAU Erlangen-Nuremberg, Erlangen, Germany; 4 Faculty of Medicine, University Hospital Erlangen, FAU Erlangen-Nuremberg, Erlangen, Germany; 5 Max-Planck-Zentrum für Physik und Medizin, Erlangen, Germany; 6 Deutsches Zentrum Immuntherapie, University Clinic Erlangen, Erlangen, Germany; 7 Institute of Medical Physics and Microtissue Engineering, FAU Erlangen-Nuremberg, Erlangen, Germany; 8 Higher Institute for Electronics and Digital Training (ISEN), Lille, France; 9 Department of Physiology, Development and Neuroscience, University of Cambridge, Cambridge, UK

**Keywords:** Nanobiotechnology, Imaging techniques

## Abstract

The glycocalyx is a complex layer of glycosylated molecules that surrounds all cells in the human body. It is involved in regulating critical cellular processes, including immune response modulation, cell adhesion and host–pathogen interactions. Despite these insights, the functional relationship between the glycocalyx architecture and cellular state has remained elusive, largely due to the structural diversity of glycocalyx constituents and their nanoscale organization. Here we show that DNA-tagged lectin labelling and metabolic oligosaccharide engineering enable multiplexed super-resolution microscopy of the glycocalyx constituents, yielding an atlas of glycocalyx architecture with nanometre resolution. Quantitative analysis of the obtained nanoscale map of glycocalyx constituents facilitates the extraction of characteristic spatial relationships that accurately report on the cellular state. We demonstrate the capacity of our approach, which we term glycan atlassing, across cell and tissue types, ranging from cultured cell lines to primary immune cells, neurons and primary patient tissue. Glycan atlassing establishes a transformative strategy for investigating glycocalyx remodelling in development and disease, potentially enabling the development of glycocalyx-centred targets in diagnosis and therapy.

## Abstract (web summary)

Multiplexed super-resolution microscopy of DNA-barcoded lectins and metabolically incorporated unnatural sugars quantitatively links the nanoscale spatial relationships of glycocalyx organization and cellular state.

## Main

**Fig. 1:**
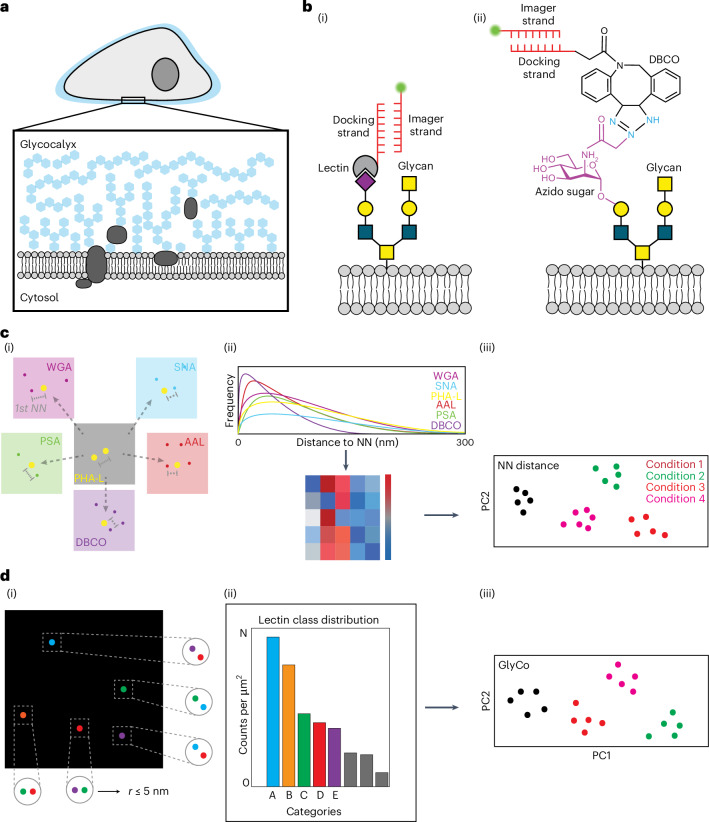
Glycan atlassing reveals the nanoscale organization of the glycocalyx. **a**, Schematic of the glycocalyx. **b**, Labelling. Schematic overview of (i) lectin labelling and (ii) metabolic labelling, targeting sialic acids. **c**, Principle of NN analysis. (i) The distance between every target location and its NN in the same and in all other channels are calculated. (ii) NN histograms of one representative channel. The colour-coded matrix reports on all inter- and intra-channel peaks of the NN distance histograms. (iii) PCA is conducted on the NN distance matrices. **d**, Principle of GlyCo. Two or more target locations from different channels are grouped if their distance is smaller than a threshold. (i) Five example classes, each containing two target locations, are shown. (ii) The most frequent classes found in a sample are collected. (iii) PCA is conducted on the NN distance matrices and the histograms from GlyCo.

The glycocalyx is a dense, dynamic layer of glycosylated molecules that surrounds all mammalian cells, making it the first part of the cell to interact with the external environment (Fig. 1a)^1,2^. It has been shown that the glycocalyx is involved in various cellular processes. These processes include, but are not limited to, immune response modulation^3^, blastocyst adhesion^4,5^ and host–pathogen interactions^6^.

In the past decades, studies have revealed numerous aspects of the functional role of the glycocalyx in cell biology. For example, interactions between sialylated ligands and immune cell siglecs were shown to reduce the immune cell activity^7^, an increase in glycocalyx thickness was observed on oncogenic events^8^, and the interplay between altered glycosylation and metastasis has been investigated^9–11^.

From a structural point of view, a key component of the glycocalyx are glycans that consist of monosaccharides such as glucose, galactose and sialic acids. The structural diversity of glycans within the glycocalyx is immense, owing to their diverse sizes, molecular compositions and stereochemical properties^12^.

A relationship between the highly complex structural organization of the glycocalyx and its biological function in health and disease seems likely but is, so far, not fully understood. Analytical methods, such as mass spectrometry, are powerful for identifying glycan structures but cannot infer details of the native spatial arrangement^13^. Mass spectrometry imaging partially addresses this drawback by ionizing glycans from the native cellular environment; however, its resolution does not allow for subcellular, let alone molecular-scale analyses^14^. Furthermore, electron microscopy has been used to study the glycocalyx^15^. Although it offers high resolution, concerns persist that its sample preparation procedures might harm the native glycocalyx structure. Moreover, electron microscopy produces only greyscale images of electron density, lacking the species-specific contrast needed to distinguish molecular components.

Optical microscopy is considered comparably non-invasive and provides species contrast^16^. However, the nanoscale architecture of the glycocalyx precludes the use of conventional optical microscopy as its resolution is limited by diffraction to ~250 nm (ref. ^17^). This changed with the advent of super-resolution microscopy methods. In particular, single-molecule super-resolution microscopy techniques rely on the temporal separation of fluorophore emission^18–20^. This approach has enabled the first analyses of glycocalyx constituents at sub-diffraction-length scales^8,21,22^. Recent work has used multiplexed dSTORM to analyse the relative abundance of glycans in different cellular organelles, although without linking this read-out to the cellular state^23^. Furthermore, the staining of the glycocalyx constituents was used to analyse diffusion metrics in the service of extracting cellular parameters from the motion of cell-surface glycans^24^. This is an interesting approach to study glycan movement on cell lines; however, its focus on tracking falls short of uncovering the organization of glycocalyx constituents. Consequently, it fails to establish a connection between spatial glycocalyx architecture and cell state. Thus, despite advances, a comprehensive quantification of glycocalyx organization and its connection to the cell state has not been achieved yet.

Here we present glycan atlassing, a quantitative mapping of cell-surface glycan architecture that unravels the interplay between nanoscale glycocalyx organization and cell state. We use lectin labelling in combination with a metabolic incorporation of unnatural sugars to tag distinct glycocalyx constituents with DNA sequences optimized for high-speed multiplexed super-resolution microscopy^25^. The obtained localization data are subsequently analysed via a tailored pipeline that extracts characteristic spatial relationships within glycan moieties, both at the level of individual and grouped localizations (GlycanConstructor^26^ (GlyCo) software package). Thus, we performed the simultaneous imaging of multiple glycan targets and the quantitative connection of their nanoscale organization to the cell state. With this approach, we establish a super-resolved atlas of the glycocalyx, connecting nanoscale glycocalyx organization and cellular state across a range of sample types—from cultured cell lines over primary immune cells and neurons and to patient tissue.

## Strategy for multiplexed DNA-PAINT microscopy of glycocalyx constituents

Glycan atlassing centrally uses DNA points accumulation for imaging in nanoscale topography (DNA-PAINT)^27^ with labelling via DNA-barcoded lectins and metabolic oligosaccharide engineering (Fig. 1b)^28^. With this integrated approach, glycan atlassing facilitates the super-resolution imaging of glycocalyx constituents. The selection of suitable lectins was based on previous studies investigating the lectin performance metrics^29^. Thus, we obtained a comprehensive overview of commercially available lectins, including their binding specificities, affinities and mutual compatibility. After the initial screening of the database, a refinement was performed using literature reporting on further experimental studies^30–32^.

Ultimately, we identified the lectins as follows: wheat germ agglutinin (WGA) binds to sialic acid and N-acetylglucosamine, which are upregulated in cancer cells to promote immune evasion and metastasis^10^. *Sambucus nigra* agglutinin (SNA) identifies α-2,6-linked sialic acids, a modification that correlates with tumour aggressiveness and immune regulation^33,34^. *Phaseolus vulgaris* leucoagglutinin detects β1-6-branched N-glycans (PHA-L), a hallmark of malignant transformation that enhances tumour invasiveness^35^. *Aleuria aurantia* lectin (AAL) binds to fucose linked (α-1,3, α-1,2, α-,4 and α-1,6) to N-acetyllactosamine, which plays roles in immune recognition and inflammatory processes^36^. *Pisum sativum* agglutinin (PSA) targets α-fucose residues, involved in pathogen recognition and immune signalling^37,38^. Lectin concentration was optimized to ensure comprehensive labelling (Supplementary Fig. 1a–e), and the control experiments verified negligible affinity of lectins to the glass coverslip (Supplementary Fig. 1f).

Simultaneously, metabolic oligosaccharide engineering was applied by incorporating N-azidoacetylmannosamine (Ac_4_ManNAz) into cell-surface sialic acid residues. The metabolically labelled sialic acids were conjugated with DNA-barcoded dibenzocyclooctyne (DBCO) via copper-free click chemistry. The strain-promoted azide–alkyne cycloaddition reaction proceeds rapidly under physiological conditions, forming stable triazole linkages between the azido-modified glycans and DBCO without the need for potentially cytotoxic copper catalysis^39^. Additionally, in the absence of azido groups, the unspecific binding of DBCO-DNA is minimal^40^. This strategy is also compatible with next-generation metabolic labelling methods using partially O-acylated unnatural azido sugars^41–43^.

## Quantitative analysis pipeline for extraction of characteristic glycan patterns

Due to the structural intricacy of the glycocalyx, the data obtained by super-resolution investigation of its architecture are inherently complex. To decipher this complexity, it is not sufficient to merely visually inspect the reconstructions. Thus, glycan atlassing uses the quantitative analysis of spatial signatures obtained by investigating nanoscale relationships between the glycan species on the cell surface.

Raw localization data are captured sequentially for each imaging target. This localization data are drift corrected and localizations for each imaging target are aligned. This is followed by the segmentation of the region of interest, excluding any non-cellular signal from the analysis. Then, clustering is performed to group multiple localizations arising from the repeated interaction between imager strands and a single docking strand. The cluster centres are taken as the target location, which we will hereafter call ‘lectin binding sites’. Subsequently, quantitative analysis (Fig. 1c,d) using nearest-neighbour (NN) peak distance matrices and grouped target locations using GlyCo is performed, followed by dimensionality reduction via principal component analysis (PCA). Supplementary Fig. 2 shows the key steps in glycan atlassing.

NN analysis is used to uncover global patterns in the spatial distribution of lectin binding sites, corresponding to parts of glycans, without further assumptions. Although NN analysis remains agnostic to the biochemical properties of the glycans, the framework of GlyCo takes into account the typical length scales of glycans known from previous studies. The identified lectin binding sites are grouped if their distance is within a 5-nm radius, assuming that at or below this threshold, they belong to the same glycan. This facilitates the reconstruction of whole glycan (sub-)structures. Regardless, both NN- and GlyCo-based analyses enable to trace how nanoscale glycan organization reflect different cellular states, facilitating biologically relevant and interpretable spatial data (see the ‘Postprocessing’ section).

## Nanoscale analysis of glycocalyx across cells and tissues

**Fig. 2:**
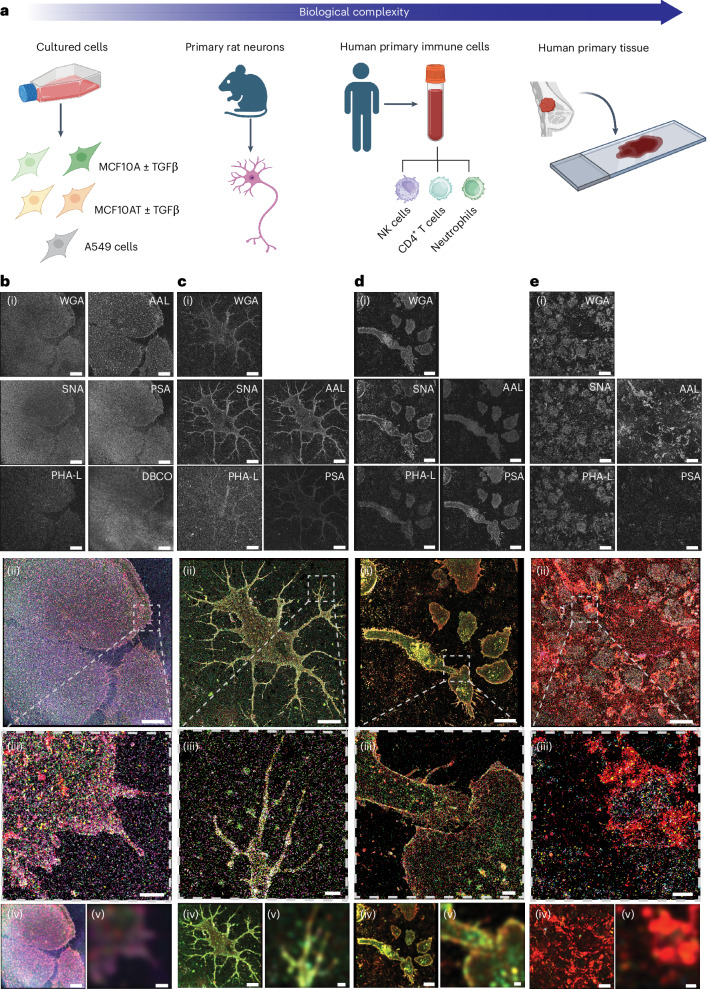
Overview of the investigated systems. **a**, Sample library investigated in this study. **b**, Cultured cells. **c**, Primary rat neurons. **d**, Human primary immune cells. **e**, Human primary tissue. (i) Individual channels depicted in greyscale. (ii) Merged channels using the following colour code: WGA, magenta; SNA, cyan; PHA-L, yellow; AAL, red; PSA, green; DBCO, purple. (iii) Zoomed-in view showing the intricate details resolved. (iv) Diffraction-limited representation of the whole field of view. (v) Diffraction-limited zoomed-in view corresponding to the data in (iii). Scale bars, 10 µm (full FoVs); 1 µm (zoomed-in views). Illustration in **a** created in BioRender; Yurekli, N. https://biorender.com/eer3k2u (2026).

Glycan atlassing was established across a range of relevant sample types of increasing complexity, ensuring that the approach was benchmarked in a controlled environment before moving to more demanding samples. The different systems studied are schematically represented in Fig. 2a. We began with standard cultured cell lines (Fig. 2b), followed by primary rat neurons (Fig. 2c), human primary immune cells (Fig. 2d) and finally primary human tissue (Fig. 2e). Each sample was labelled and analysed using the same protocol, which was only slightly adapted to satisfy specific requirements of the systems studied (Methods). Additional examples are shown in Supplementary Fig. 3.

In brief, the samples were incubated with DNA-barcoded lectins. For MCF10A/AT cells, they were also incubated with Ac_4_ManNAz for the metabolic labelling of sialic acids, followed by copper-free click chemistry to attach the docking strand. The samples were then subjected to multiplexed DNA-PAINT microscopy (Supplementary Fig. 4 shows the quality metrics), data analysis and quantitative spatial mapping of the obtained high-dimensional nanoscale imaging data.

With this approach, we gradually moved from in vitro to ex vivo, achieving a robust demonstration of glycan atlassing across cells and tissues. For each sample type shown in Fig. 2b–e, reconstructions from individual imaging rounds targeting different glycan species are presented in greyscale alongside the respective labelling agent. Full fields of view (FoVs) and zoomed-in views are shown alongside diffraction-limited images, highlighting the substantial gain in resolution and the successful visualization of the nanoscale glycocalyx structure. In particular, differences between lectins are evident at the qualitative level depicted here, as well as differences between identical lectins on different sample types. This indicates that our approach has target- and sample-specific read-out potential, even at the qualitative level, which we extend to the quantitative level.

## Glycan atlassing enables tracing of cancer progression

**Fig. 3:**
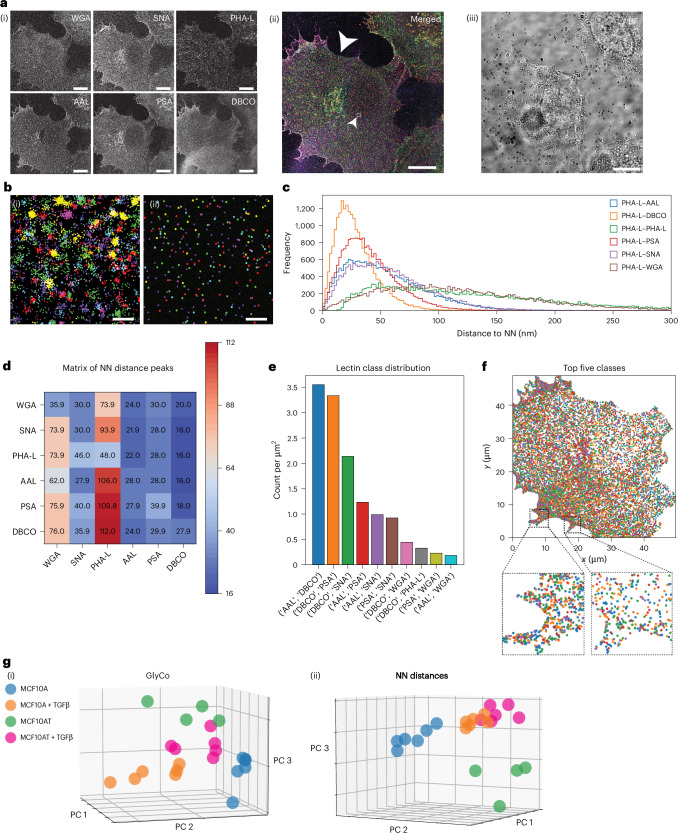
Glycan atlassing traces early oncogenic events. **a**, (i) Individual channels in greyscale. (ii) Merged data of individual channels (the white arrowhead indicates the cell analysed here; Fig. 2 shows the colour map). (iii) FoV in bright field (BF). The inset with the small arrowhead denotes the area represented in **b**. **b**, Representative example of clustering localization clouds to single target locations. (i) Raw localizations. (ii) Clustered localizations. **c**, NN histograms of one representative channel (relationship of PHA-L to all channels). **d**, Maxima from NN histograms across all comparisons. **e**, Lectin class distribution from GlyCo. The ten most frequent classes and their spatial densities are shown. **f**, Spatial arrangement of the classes shown in **e**. **g**, PCA plots for (i) GlyCo and (ii) NN peak distances for all the samples investigated, comparing the four stages of EMT modelled. Scale bars, 10 µm (**a**); 100 nm (**b**). For each cell type, data are collected from at least two seedings. Source data

As the first imaging target, we chose immortalized mammary epithelial cells (MCF10A). In addition, a transformed variant (MCF10AT), constitutively expressing the Harvey rat sarcoma viral oncogene was included. Both cell lines were either treated with the tumour growth factor β (TGFβ) or left untreated. Thus, we investigated the panels of MCF10A, MCF10A + TGFβ, MCF10AT and MCF10AT + TGFβ as a model system for epithelial-to-mesenchymal transition (EMT), a key step in cancer progression^44,45^. In combination with lectin labelling, we performed the metabolic labelling of terminal sialic acids on these cells (Fig. 3a,b). Additional data for cells in the MCF10A panel are shown in Supplementary Fig. 5.

The data reveal distinct variations in local density within individual imaging targets, alongside global differences across targets (Fig. 3c–f). Furthermore, the spatial distributions of different sialic acid and fucose linkages can be observed at nanometre resolution. Finally, and most strikingly, PCA (Fig. 3g) of the multidimensional datasets from (i) GlyCo and (ii) NN peak distribution are able to faithfully separate the different stages of cancer progression within the MCF10A-based cellular model system.

To characterize how the PCA-based separation is achieved, we calculated the loading vectors of the principal components. These loadings quantify the correlation between each original variable (for example, specific NN distances or GlyCo cluster frequencies) and the principal components. High absolute loading values indicate a strong contribution to that component (Supplementary Fig. 6). This analysis yielded that there is no single alteration in glycan organization that explains the observed shifts; rather, the collective redistribution is relevant.

It is worth pointing out that the clusters of TGFβ-stimulated cells are located closer to each other than the unstimulated conditions. This indicates that TGFβ stimulation drives both MCF10A and MCF10AT cells to a closely related hyperproliferative phenotype, which is mirrored in the glycocalyx state. Taken together, the characteristic nanoscale glycocalyx signatures accurately report on the internal cellular state, and glycan atlassing is able to retrieve this information.

## Glycan atlassing enables analysis of neuronal glycosylation

**Fig. 4:**
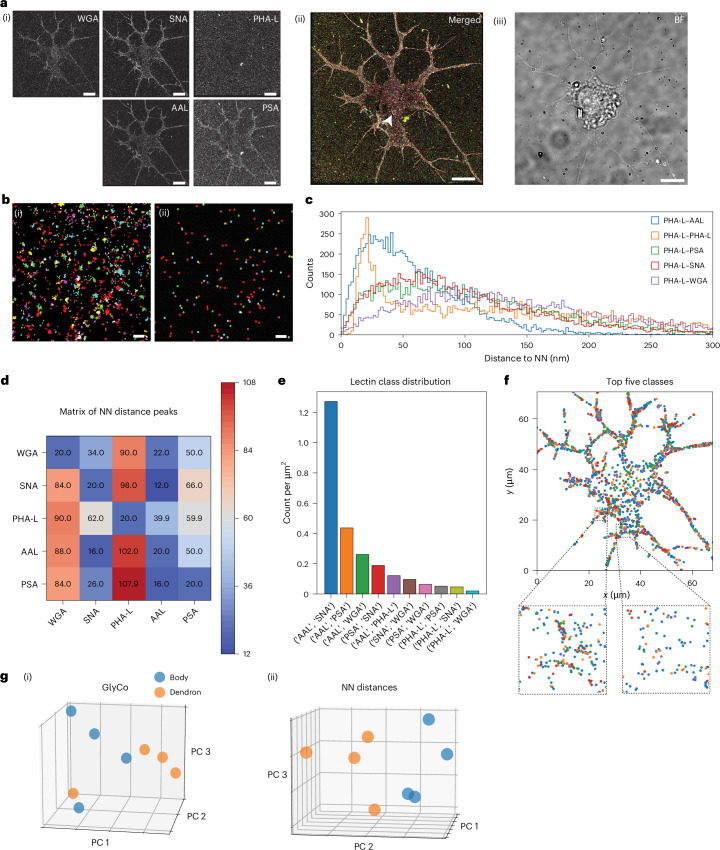
Glycan atlassing reveals characteristic neuronal glycosylation patterns. **a**, (i) Individual channels in greyscale. (ii) Merged data of individual channels (Fig. 2 shows the colour map). (iii) FoV in bright field. The inset with the small arrowhead denotes the area represented in **b**. **b**, Representative example of clustering localization clouds to single target locations. (i) Raw localizations. (ii) Clustered localizations. **c**, NN histograms of one representative channel (relationship of PHA-L to all channels). **d**, Maxima from NN histograms across all comparisons. **e**, Lectin class distribution from GlyCo. The ten most frequent classes and their spatial densities are shown. **f**, Spatial arrangement of the classes shown in **e**. **g**, PCA plots for (i) GlyCo and (ii) NN peak distances for all the samples investigated, comparing the characteristic glycosylation patterns between the neuron cell body and dendrons. Scale bars, 10 µm (**a**); 100 nm (**b**). Data are collected from at least two seedings. Source data

Next, we proceeded to use glycan atlassing on embryonic rat hippocampal neurons (Fig. 4). Primary neurons were extracted from embryonic rat hippocampal tissue (Methods). Additional data for neurons used in the study are shown in Supplementary Fig. 7.

Interestingly, PHA-L signal is low in the neurons investigated, consistent with the reported timing of complex N-glycosylation (the labelling target of PHA-L) in the primary neuronal culture. Thus, this observation supports our analysis, as elaborate N-glycosylation is expected to become prominent in matured primary neurons, whereas the cells investigated here are considered in an early stage of development^46^. As before, glycan atlassing is able to distinguish between the analysed conditions based on characteristic glycan patterns.

Thus, glycan atlassing reveals differential glycosylation signatures within individual neurons, which may indicate an unidentified regulatory axis. For example, glycosylated proteins may engage with galectins, modulating the membrane receptor residence times and internalization kinetics, as previously reported^47^.

## Glycan atlassing identifies immune cell state

**Fig. 5:**
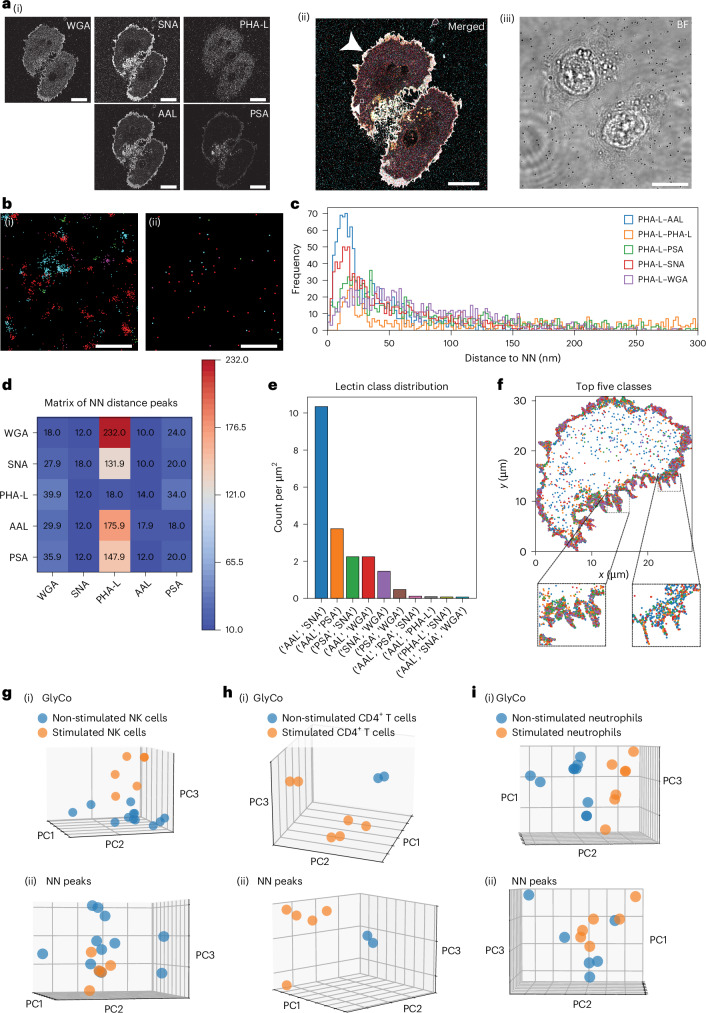
Glycan atlassing traces immune cell activation. **a**, (i) Individual channels in greyscale. (ii) Merged data of individual channels (the white arrowhead indicates the cell analysed here; Fig. 2 shows the colour map). The inset with the small arrowhead denotes the area represented in **b**. (iii) FoV in bright field. **b**, Representative example of clustering localization clouds to single target locations. (i) Raw localizations. (ii) Clustered localizations. **c**, NN histograms of one representative channel (relationship of PHA-L to all channels). **d**, Maxima from NN histograms across all comparisons. **e**, Lectin class distribution from GlyCo. The ten most frequent classes and their spatial densities are shown. **f**, Spatial arrangement of the classes shown in **e**. **g**–**i**, PCA plots for (i) GlyCo and (ii) NN peak distances for all the samples investigated, comparing the characteristic glycosylation patterns between activated and non-activated NK (**g**), CD4^+^ T cells (**h**) and neutrophils (**i**). *n* = 4 independent isolations, two cells per staining for NK cells, *n* = 1 isolation, two independent seedings and stainings, two cells per staining for CD4^+^ T cells; *n* = 2 isolations, two independent seedings and stainings, one cell per staining for neutrophils. Scale bars, 10 µm (**a**); 100 nm (**b**). Source data

To investigate the functional glycocalyx architecture of immune cells in different cellular states, we next performed glycan atlassing on a range of different primary immune cells (Fig. 5). In particular, we investigated primary natural killer (NK) cells, primary CD4^+^ T cells and primary neutrophils. Additional data for primary immune cells used in the study are shown in Supplementary Fig. 8.

NK cells, vital for innate immunity, exhibit rapid cytotoxic responses without de novo glycan or protein biosynthesis for acute actions^48,49^. Our method revealed a profound and rapid nanoscale remodelling of the NK cell glycocalyx within 5 min of incubation with A549 target cells. In particular, areas with distinctly increased and depleted signal are present, highlighting the extraction of spatial heterogeneity at the relevant scales. The observed swift reorganization is difficult to explain by changes in biosynthesis and is probably driven by faster mechanisms^50^. One of these might be the exocytosis of cytotoxic granules^51,52^. Fusion of these granules with the cell membrane probably rapidly alters the surface glycan patterns^53^. This rapid membrane merging and the insertion of glycosylated exocytosome components could centrally contribute to the observed changes in surface glycosylation^54^.

Furthermore, lateral redistribution and clustering might play a role. Rapid activation signals can induce the lateral redistribution or clustering of specific glycoconjugates, driven by protein–protein interactions, lipid microdomain dynamics or cytoskeletal rearrangements, altering nanoscale patterns^55^. Enzymatic modifications on the cell surface may also contribute^56^. Cell-surface glycosidases can rapidly modify existing glycan structures, swiftly altering glycan epitopes and spatial relationships^57^. Last, the shedding or uptake of glycoconjugates can lead to swift changes in the cell-surface glycome^58^. This selective removal or acquisition of glycans probably contributes to the observed rapid remodelling^59^.

Glycan atlassing enables the precise identification of the activation state of NK cells, CD4^+^ T cells and neutrophils within the short time frame of immune cell activation (for example, 5 min in case of NK cells) used in this study. Earlier studies have suggested that the full turnover of the cell-surface glycome occurs within a relatively longer period of 24–48 h (ref. ^60^). However, these studies focused on the global turnover of the glycocalyx bulk, not on more subtle structural changes.

Our analysis suggests that immune cells are capable of rapid adaptations in their cell-surface glycosylation patterns in response to external stimuli. This indicates that the glycocalyx is remarkably dynamic in the timescale of minutes and highly responsive to the cellular environment, displaying distinct and clearly separatable glycan patterns to the outside.

## Glycan atlassing differentiates healthy and tumour tissues

Next, we used glycan atlassing on primary patient tissue slices. We investigated primary breast adenocarcinoma tissue, with tumour regions and surrounding non-tumour regions present in the same slice, allowing for internal referencing. The categorization into tumour and non-tumour regions was performed by histopathological grading.

**Fig. 6:**
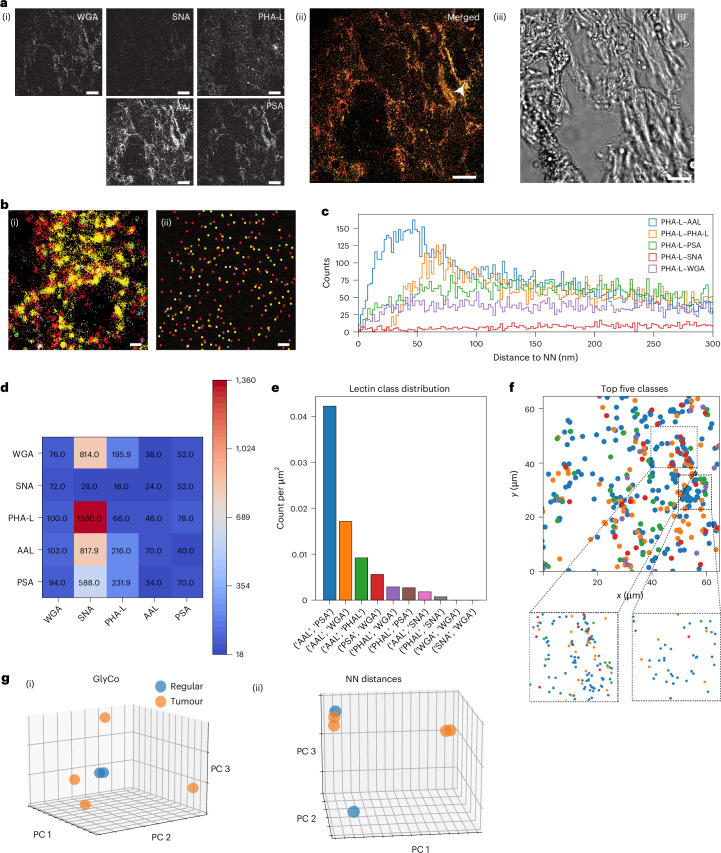
Glycan atlassing separates healthy and tumour tissue. **a**, (i) Individual channels in greyscale. (ii) Merged data of individual channels (the white arrowhead indicates the cell analysed here; Fig. 2 shows the colour map). The inset with the small arrowhead denotes the area represented in **b**. (iii) FoV in bright field. **b**, Representative example of clustering localization clouds to single target locations. (i) Raw localizations. (ii) Clustered localizations. **c**, NN histograms of one representative channel (relationship of PHA-L to all channels). **d**, Maxima from NN histograms across all comparisons. **e**, Lectin class distribution from GlyCo. The ten most frequent classes and their spatial densities are shown. **f**, Spatial arrangement of the classes shown in **e**. **g**, PCA plots for (i) GlyCo and (ii) NN peak distances for all the samples investigated, comparing the characteristic glycosylation patterns between tumour and non-tumour areas. Scale bars, 10 µm (**a**); 100 nm (**b**). Data are collected from two tissue slices. Source data

Figure 6 shows the results of glycan atlassing the panel of primary patient tissue slices. We note that since the cutting of the tissue passes through cells, some of the signal in this case might originate from glycans that are not yet incorporated into the glycocalyx, residing, for example, still in the golgi apparatus. In service of minimal sample preparation time for potential future applications, we decided to not exclude this potential origin and analyse the total signal from the imaged slice. Additional data for the tissue sections are shown in Supplementary Fig. 9.

Indeed, the PCA-based investigation enabled the identification of tumour and non-tumour regions by analysing their characteristic nanoscale glycan signatures. Interestingly, non-tumour regions form a tight cluster in the PCA whereas tumour regions are more spread out, suggesting a more heterogeneous glycocalyx landscape, consistent with the failed regulation of biosynthetic pathways in cancer cells^61^.

## Conclusion

Here we report on glycan atlassing, comprehensively enabling the characterization of cellular state by tracing the nanoscale glycocalyx architecture. Glycan atlassing integrates DNA-PAINT, bioorthogonal chemistry and lectin-based glycan targeting, as well as quantitative analysis. Beyond establishing the methodology and demonstrating it on a range of relevant biological systems—from cultured cell lines over primary cells to patient tissue—we identify several so-far-unknown processes that functionally link the glycocalyx state to the cell state.

In particular, our results indicate that EMT strongly modulates the glycocalyx structure. In light of earlier studies by our group and others suggesting a functional link between the cancer glycocalyx and cancer progression, glycan atlassing will probably be a highly valuable approach to characterize the glycocalyx state in fundamental cancer biology^8,62^. Our results further suggest differential glycosylation states at the subcellular level in primary neurons, raising the question about how neuronal activity in health and disease is modulated by the nanoscale architecture of the neuronal glycocalyx. Strikingly, we observed the rapid reorganization of specific glycosylation patterns in response to immune cell activation on NK cells, CD4^+^ T cells and neutrophils. This result points towards a hitherto uncharacterized axis of dynamic glycocalyx adaptation in immune regulation, with profound implications for glyco-immunology and cell-based therapies. Finally, we were able to take steps towards identifying tumour tissues based on characteristic glycan patterns. Our observations align well with previous studies reporting aberrant fucosylation and fucosyltransferases in cancer cells^63,64^. Going beyond such previous studies that identified bulk changes in tumour cell glycosylation, our approach allows for the direct characterization of glycosylation at the nanoscale level down to distinct glycan linkages. Understanding the precise organization of these glycocalyx structures will probably contribute to a better annotation of cancer cell state and—in a more clinical setting—may help in the development of targeted diagnostic and therapeutic strategies. Glycan atlassing has a transformative potential for implementation in diagnostic routines in clinical settings, for example, by classifying tumours according to their functional glycocalyx fingerprint. This is especially relevant considering the emerging field of immune cell regulation mediated by the Siglec–sialome axis^65,66^.

Despite these advances, glycan atlassing as presented here has limitations. First, a critical barrier that needs to be broken is the limited availability of specific glycan-labelling agents. Compared with the labelling tools available for proteins or genomic sequences, glycan-targeted labelling is currently less developed. Progress in this area is needed and will immediately expand the scope of glycan atlassing. Second, our study is currently limited to six labelled species. Recent reports have demonstrated a larger number of accessible labelling targets^67^, and the implementation of such strategies will be certainly valuable for the future development of glycan atlassing. We note, however, that the experimental complexity and the time demand scales with the number of imaged species. Thus, any implementation should balance feasibility with scope. On the basis of the results presented here, we are confident that the current number of imaging targets enables the extraction of meaningful nanoscale glycocalyx signatures. If fewer species are considered, the separation power decreases; thus, the chosen number is suggested to be reasonable (Supplementary Fig. 10). Third, although glycan atlassing is compatible with clinical samples, its current implementation requires a high level of expertise and hands-on time from the experimenter, which probably precludes broad clinical adaptation. Here innovation in data acquisition and analysis automation will be of profound importance. Finally, the analysis of NN distances currently only considers the peak positions of the extracted distributions, essentially projecting the histogram onto a single value used as input for further analysis. More advanced approaches could probably extract more refined information from the datasets. To test this, we ran PCA on the full NN distance distributions, which yielded good separation between conditions (Supplementary Fig. 11). However, it is noteworthy that despite this reduction in dataset complexity, precise feature extraction is still possible, suggesting the high robustness of our approach.

From an experimental perspective, Glyco-STORM^23^ will be an appropriate method to image organelle glycosylation. For the tracking of glycan dynamics, Lectin-PAINT^24^ seems recommendable, and glycan atlassing, presented here, is the method of choice for the nanoscale analysis of glycan architecture and its relationship to the cell state. In addition, if single-sugar resolution is required, our recently published method relying on resolution enhancement via sequential imaging should be considered^40^.

In conclusion, glycan atlassing provides a comprehensive method for characterizing cellular states by analysing the nanoscale architecture of the glycocalyx and distinguishing cellular states across different biological systems through the identification of characteristic glycan patterns. The ability to quantify and visualize glycocalyx signatures at the nanometre scale offers considerable potential to advance our understanding of cellular behaviour and to develop targeted therapies. Glycan atlassing is, therefore, a powerful tool in fundamental glycobiology with the potential to drive innovation in biomedical research and clinical applications.

## Methods

### Ethics statement

The research complies with all ethical regulations. Immune cell isolation from human blood and analysis of human primary tissue was approved by the ethics committee of the University Clinic Erlangen (reference numbers 22-321-Bp and 24-349-Bp).

### Cell culture

MCF10A/AT cells were cultured in T-25 flasks (Greiner Bio-one, catalogue number (cat. no.) 690175) containing Dulbecco’s modified Eagle’s medium/F12 (Gibco, reference number (ref. no.) 21041-025) medium supplemented with 5% horse serum (Gibco, ref. no. 16050-112), 20 ng ml^−1^ of epidermal growth factor (Gibco, ref. no. PHG0311), 0.5 μg ml^−1^ of hydrocortisone (Sigma-Aldrich, ref. no. H0396), 100 ng ml^−1^ of cholera toxin (Sigma-Aldrich, cat. no. C8052-5MG), 10 μ ml^−1^ of insulin (Sigma-Aldrich, cat. no. I1882-100MG) and 1% penicillin–streptomycin (Sigma-Aldrich, cat. no. P0781-100ML). Cells were maintained at 37 °C in a humidified atmosphere with 5% CO_2_. On reaching 70%–80% confluency, cells were washed with 5 ml of Ca^2+^/Mg^2+^-free Dulbecco’s phosphate-buffered saline (DPBS) (Gibco, ref. no. 14190-094) and harvested by enzymatic dissociation by brief incubation with 0.05% trypsin ethylenediaminetetraacetic acid (EDTA) solution (Gibco, ref. no. 2500-054) for 8–10 min. Following trypsin neutralization with complete medium, cells were centrifuged at 3,000*g* for 5 min, and the resulting pellet was resuspended in 5 ml of complete growth medium. For experimental assays, cells were seeded into eight-well chamber slides (ibidi, ref. no. 80807-90) at a 1:10 dilution and incubated for 24 h prior sample preparation. For the TGFβ-treated variants of MCF10A/AT, cells were cultured in media containing 5 ng ml^−1^ of TGFβ (Bio-Rad, ref. no. PHP143B) for four subsequent passages. Then, the cells were continued in culture using complete media without TGFβ.

A549 cells were cultured in T-25 flasks containing RPMI-1640 medium (Gibco, ref. no. 31870-025) supplemented with 10% fetal bovine serum (Gibco, ref. no. A31605-02), 1% penicillin–streptomycin and 1% GlutaMAX (Gibco, ref. no. 35050-38). Cells were maintained at 37 °C in a humidified atmosphere with 5% CO_2_. On reaching 70%–80% confluency, cells were washed with 5 ml of Ca^2+^/Mg^2+^-free DPBS and harvested by enzymatic dissociation by brief incubation with 0.05% trypsin EDTA solution for 8–10 min. Following trypsin neutralization with the complete medium, cells were centrifuged at 3,000*g* for 5 min, and the resulting pellet was resuspended in 5 ml of complete growth medium. For co-culture experiments, cells were seeded into eight-well chamber slides at a 1:10 dilution and incubated for 24 h before experimental manipulations.

### Isolation and expansion as well as sample preparation of primary NK cells

NK cells were isolated from peripheral blood using the NK cell isolation kit (Stem Cell Technologies, cat. no. 19665) following the manufacturer’s instructions, which involves density gradient centrifugation and magnetic bead separation. The isolated NK cells were then expanded using the NK cell expansion kit (Stem Cell Technologies, cat. no. 100-0711), which includes the base medium (Stem Cell Technologies, cat. no. 100-0712), supplement (Stem Cell Technologies, cat. no. 100-0715) and coating material (Stem Cell Technologies, cat. no. 100-0714). Briefly, a 24-well plate (Stem Cell Technologies, cat. no. 38044) was coated with the coating material and incubated for 2 h at room temperature and then rinsed with Ca^2+^/Mg^2+^-free DPBS. Isolated NK cells were seeded onto the coated plate at a density of 1 × 10^6^ cells ml^−1^ in ImmunoCult NK Cell Expansion Medium and incubated at 37 °C and 5% CO_2_. On day 3 or 4, an additional expansion medium was added, and the cells were further incubated. On days 7 and 10/11, the cells were harvested via Ca^2+^/Mg^2+^-free DPBS and reseeded onto coated eight-well plates (2 × 10^5^ cells μgml^−1^) in a fresh expansion medium and incubated for 72 h to prepare the samples for fixation.

### Co-culture of NK cells and A549 cells

A549 cells were seeded at a 1:10 concentration onto NK-cell-surface-coated eight-well plates and incubated at 37 °C in a humidified atmosphere containing 5% CO_2_ for 24 h to allow for proper adherence and growth. Separately, previously seeded and expanded NK cells were resuspended for a passaging and co-culture experiment. For co-culture experiments, resuspended cells in fresh media were subsequently added to the A549 cells in a concentration of 2 × 10^5^ cells ml^−1^. The co-culture was incubated at 37 °C in 5% CO_2_ for a duration of 5 min, during which NK cells adhered to the A549 cancer cells. The interaction between NK and A549 cells was closely monitored using light microscopy. Once the physical interactions between NK and A549 cells were observed, the samples were immediately fixed.

### Preparation of tissue slices

Samples were obtained from the Central Biobank of the University Clinic Erlangen. Cryosections were fixed using 4% paraformaldehyde for 20 min and transferred to #1.5 glass slides. Before imaging, a chamber (ibidi sticky slides, ref. no. 80427) was placed on top of the glass slides and filled with PBS to facilitate staining, imager strand addition and washing steps.

### Metabolic incorporation of Ac4ManNAz and copper-free click chemistry

For the metabolic labelling of sialic acids, the MCF10A/T panel was seeded into slides, as described above. After 3 h, the cell culture medium is supplemented with 50 µM of Ac_4_ManNAz and incubated for 72 h. After incubation, cells were washed twice with Ca^2+^/Mg^2+^-free DPBS and then refreshed with standard growth medium supplemented containing 50 µM of DBCO conjugated to docking strand R6 for 2 h in the incubator to facilitate the click reaction. Then, cells were fixed and stained with lectins.

### Primary neuronal cultures

Hippocampi of E18 Sprague Dawley rats (Transnetyx, SKU number SDEHP) were washed thrice in ice-cold Hanks’s balanced salt solution (Thermo Fisher, cat. no. 14175095) with 1% penicillin–streptomycin (Thermo Fisher, cat. no. 15140122). Tissue was then incubated for 10 min in 0.05% trypsin EDTA (Gibco, cat. no. 15400054) at 37 °C. Trypsin was removed and the tissue was washed ten times in prewarmed Hanks’s balanced salt solution with 1% penicillin–streptomycin. Hanks’s balanced salt solution was replaced by 1 ml of warm Neurobasal Medium (Thermo Fisher, cat. no. 12348017) supplemented with 1% penicillin–streptomycin, 1% GlutaMAX (Thermo Fisher, cat. no. 35050061) and 2% B27 (Thermo Fisher, cat. no. 1750404). Cells were then mechanically dissociated by pipetting up and down about 30 times with a 200-µl pipette. Also, 100,000–150,000 cells were seeded on glass-bottomed Petri dishes (WPI, cat. no. FD35-100) coated with 10 µg ml^−1^ of poly-D-lysine (Sigma-Aldrich cat. no. p6407-5mg) and 1 µg ml^−1^ of laminin (Merck, cat. no. L2020-1MG) in 2 ml of Neurobasal Medium supplemented with 1% penicillin–streptomycin, 1% GlutaMAX and 2% B27. The medium was replaced the next day, and afterwards, half of the medium was replaced every 3 days.

### Isolation, activation and preparation of primary human CD4^+^ T cells

CD4^+^ T cells were isolated from blood samples of healthy donors. First, peripheral blood mononuclear cells were collected after Ficoll gradient isolation (Ficoll-Paque PLUS, VWR). CD4^+^ T cells were then separated using commercially available CD4^+^ T cell isolation kit for human samples (Miltenyi), according to manufacturer’s instructions. Here 0.5 × 10^6^ CD4^+^ cells were resuspended in Iscove’s modified Dulbecco’s medium (Gibco) supplemented with 10% fetal bovine serum (PanBiotech) and 1% penicillin–streptomycin (Gibco). To activate the cells, anti-human CD3 (α-CD3, 1 µg ml^−1^, Ultra-LEAF Purified anti-human CD3 Antibody, BioLegend) together with anti-human CD28 (α-CD28, 2 µg ml^−1^, Ultra-LEAF Purified anti-human CD28 Antibody, BioLegend) and recombinant human IL-2 (20 ng ml^−1^, ImmunoTools) were also provided. The cells were cultured for 3 days at 37 °C and 5% CO_2_. For control, non-stimulated and freshly isolated CD4^+^ T cells from the same donor were used. Samples were then fixed using 4% paraformaldehyde for 10 min at room temperature and washed three times with PBS before lectin labelling.

### Isolation, stimulation and preparation of primary human neutrophils

Human neutrophils were isolated using a commercially available kit (MACSxpress Whole Blood Neutrophil Isolation Kit, Miltenyi), according to the manufacturer’s instructions. Then, 10^6^ neutrophils were prepared in RPMI-1640 medium (Gibco) supplemented with 10% fetal bovine serum (PanBiotech) and 1% penicillin–streptomycin (Gibco). For stimulation conditions, recombinant human tumour necrosis factor-alpha (100 ng ml^−1^, ImmunoTools) was also added and the cells were cultured for 2 h at 37 °C and 5% CO_2_. Non-stimulated neutrophils were kept in the same conditions for culture, but without tumour necrosis factor-alpha. Samples were fixed using 4% paraformaldehyde for 10 min at room temperature and washed three times with PBS before lectin labelling.

### Sample fixation

Samples were washed with DPBS and Ca^2+^/Mg^2+^ (Gibco, ref. no. 14040-091) three times. Then, 4% paraformaldehyde, diluted from a 16% stock solution (Thermo Scientific, ref. no. 28908) to 4% working solution via Ca^2+^/Mg^2+^-free DPBS was added to wells and cells were incubated at room temperature for 15 min. Then, cells were washed three times with Ca^2+^/Mg^2+^-free DPBS. After fixation, cells were permeabilized with 0.1% Triton X (Alfa Aesar, cat. no. A16046) for 10 min at room temperature, followed by four DPBS and Ca^2+^/Mg^2+^-free washing steps.

### Lectin labelling

A lectin cocktail (2.5 μg μg ml^−1^ of each lectin) prepared in 1× Tris buffer (Fisher Bioreagents, ref. no. M-15836) was applied to the cells at room temperature for 30 min, allowing specific binding to glycan targets on the cellular surface. Following incubation, cells were washed three times with Ca^2+^/Mg^2+^-free DPBS. For the MCF10A panel cells, pre-permeabilization is performed using 0.1% Triton X-100 for 10 min at room temperature, followed by four washing steps with Ca^2+^/Mg^2+^-free DPBS and incubation with lectins as above. Before imaging, another permeabilization step using 0.1% Triton X-100 was performed for 10 min at room temperature, followed by four washing steps with Ca^2+^/Mg^2+^-free DPBS. For primary neurons, a permeabilization step using 0.1% Triton X-100 was performed for 10 min at room temperature, followed by four washing steps with Ca^2+^/Mg^2+^-free DPBS before imaging. For primary immune cells and tissue sections, samples were permeabilized with 0.2% Triton X-100 for 10 min at room temperature, followed by four washing steps with Ca^2+^/Mg^2+^-free DPBS. To test the effect of fixation, we inverted the protocol sequence and tested live-cell staining with WGA, followed by fixation. No detectable difference was observed (Supplementary Fig. 12).

### Optical setup

DNA-PAINT imaging was carried out on an inverted microscope (Nikon Instruments, Eclipse Ti2) with the Perfect Focus System. Objective-based total internal reflection fluorescence (TIRF) mode was used, using a high-numerical-aperture objective (Nikon Instruments, Apo SR TIRF ×100, numerical aperture of 1.49, oil) and the Nikon TIRF module. A 560-nm laser (MPB Communications, 1 W) was used for excitation and coupled into the microscope via the TIRF module. The power of the laser beam was controlled in free space using a filter wheel (Thorlabs, FW212CNEB). The laser beam was passed through a clean-up filter (Chroma Technology, ZET561/10) and coupled into the microscope objective using a beamsplitter (Chroma Technology, ZT561RDC). Fluorescence was spectrally filtered using an emission filter (Chroma Technology, ET600/50m, and ET575LP) and imaged on a scientific complementary metal–oxide–semiconductor camera (Hamamatsu Orca Fusion) without further magnification, resulting in an effective pixel size of 130 nm after 2 × 2 binning. TIRF illumination was used for all the measurements with a laser power of ~33 mW above the objective. The central area of 1,152 × 1,152 pixel^2^ (576 × 576 after binning) of the camera was used as the region of interest. Raw microscopy data were acquired using μManager (v. 2.0.3).

### DNA sequences

Docking and imager strand sequences are previously optimized for orthogonal binding specificity. The sequences used for DNA-PAINT were as follows:

R1 (WGA) with 5 × R1 sequence TCCTCCTCCTCCTCCTCCT

R2 (SNA) with 5 × R2 sequence ACCACCACCACCACCA

R3 (PHA-L) with 7 × R3 sequence CTCTCTCTCTCTCTCTCTC

R4 (AAL) with 7 × R4 sequence ACACACACACACACA

R5 (PSA) with 5 × R5 sequence CTTCTTCTTCTTCTT

R6 (DBCO) with 5 × R6 sequence AACAACAACAACAACAA.

All DNA oligonucleotides were purified via high-performance liquid chromatography and obtained from Metabion.

### Imager strand preparation for DNA-PAINT imaging

Imaging buffer was prepared by combining 50 ml of Ca^2+^/Mg^2+^-free DPBS with 0.0146 g of EDTA (PanReac, cat. no. 60-00-4), 1.461 g of sodium chloride (Alfa Aesar, cat. no. A12313), and 10 µl of Tween-20 (MP Biomedicals, cat. no. 103368) in a 50-ml Falcon tube. All the components were thoroughly mixed until completely dissolved. The prepared buffer was stored at 4 °C until use in subsequent imager strand preparation. Imaging strands for DNA-PAINT were prepared by diluting 1 µM of the stock solutions in the buffer, the optimal imager concentration to achieve sparse blinking ranged from 0.075 to 0.5 nM, adjusted for sample types and targets, focusing on sparse single-molecule signals. Samples were incubated with a 1:3 dilution of 90-nm gold nanoparticles (Absource, cat. no. CG-90-20), which were used as fiducial markers for drift correction and channel alignment.

### Multiplexed DNA-PAINT

DNA-PAINT imaging was conducted via six subsequent imaging rounds with only one imager type in each round. For each target, 20,000 frames of single-molecule blinks are captured with a frame time of 100 ms. A laser power of ~33 mW at the objective was used. Between imaging rounds, thorough washing for at least four times with Ca^2+^/Mg^2+^-free DPBS was performed, ensuring no residual signal from the previous imager solution remained before introducing the next imager solution.

### Number of samples

For the MCF10A panel, data from two independent seedings are shown. Each seeding was staining independently. Data from at least two cells per staining are shown. For neurons, data from two independent seedings are shown. Each seeding was staining independently. Data from at least one cell per staining are shown. For NK cells, data from four independent isolations are shown. Each isolation was stained independently. Data from at least two cells per staining are shown. For T cells, data from one isolation are shown. Isolated cells were stained in two independent samples. Data from at least two cells per staining are shown. For neutrophils, data from two isolations are shown. Each isolation was stained in two independent samples. Data from at least one cell per staining are shown. For tissue sections, data from two independent slices are shown. Each slice was stained in two independent samples. Data from at least two FoVs are shown.

### Statistics and reproducibility

No statistical methods were used to predetermine the sample sizes. No data were excluded from the analyses. The investigators were not blinded to allocation during experiments and outcome assessment.

### Postprocessing

Raw fluorescence data were reconstructed using the Picasso software package^27^ (v. 0.7.4). For identifying distinct blinks in each frame, an intensity threshold of 5,000 was used. Drift correction was performed using redundant cross-correlation, followed by precise fiducial-based drift correction with gold nanoparticles. The six channels were aligned using gold nanoparticles as fiducial markers. The region of interest is segmented using the polygon pick function from Picasso (no signal from outside the cell is included in the analysis). All downstream analysis after segmentation until the calculation of cluster centres was performed using a custom version of Picasso in Python. An estimate of experimental localization precision is calculated for each individual channel of images using NN-based analysis^68^. For clustering, a radius of two times the experimental NN-based analysis precision was used. The minimum number of localizations inside a cluster was two. Time signatures of blinking events inside each cluster were analysed to account for unspecific sticking events. For a single cluster, if a time bin of 200 frames (1% of the total length of the stack) contains more than 90% of all the events in that cluster, the respective cluster is rejected. Thus, single and/or atypically extended events are rejected as false localization. Cluster centres are calculated and are used as the location of glycan targets.

For further analysis, two approaches were used. First, we calculated the NN distances across all glycan localizations, both within channels and between different channels. Histograms of the distances to the first NN were plotted and the peak of the distributions were collected in a 5 × 5 matrix (6 × 6 for datasets with DBCO). Second, we performed a classification of lectin binding sites that are frequently occurring in close spatial proximity within the same channel and across channels using a cut-off radius of 5 nm. Each observed combination of two or more lectin binding sites was assigned a different class. The distribution of classes per square micrometre was plotted and the cellular location of these classes was mapped. To handle these multidimensional data, we performed standard linear dimensionality reduction using PCA in Python.

### Reporting summary

Further information on research design is available in the Nature Portfolio Reporting Summary linked to this article.

## Online content

Any methods, additional references, Nature Portfolio reporting summaries, source data, extended data, supplementary information, acknowledgements, peer review information; details of author contributions and competing interests; and statements of data and code availability are available at 10.1038/s41565-026-02151-y.

## Supplementary information

Supplementary Information Supplementary Figs. 1–12.

Reporting Summary

## Source data

Source Data Figs. 3–6 Lectin class data and NN distance data.

## Data Availability

All raw data are available via Zenodo at 10.5281/zenodo.16739131 (ref. ^69^). Source data are provided with this paper.

## Code availability

Analysis code can be downloaded from GitHub (http://github.com/moecklgroup/DNAPaint_nNAnalysis)^26^.
