# Supplementary material for: Glycan atlassing enables functional tracing of cell state
Source: Nat Nanotechnol. 2026 May 14;21(5):720–31. doi: 10.1038/s41565-026-02151-y (PMC13186696; doi:10.1038/s41565-026-02151-y)
Supplement: Supplementary file 1 — Supplementary Figs. 1–12. [file 41565_2026_2151_MOESM1_ESM.pdf]

nature nanotechnology

Supplementary information https://doi.org/10.1038/s41565-026-02151-y

Glycan atlassing enables functional tracing of cell state

In the format provided by the authors and unedited

Table of contents page

Figure S1: Control experiments for labeling optimization	2
Figure S2: Experimental flow of Glycan Atlassing	3
Figure S3: Additional reconstructions across sample types	4
Figure S4. Quality metrics	5
Figure S5: Further data on MCF10A panel	8
Figure S6: PCA loadings	12
Figure S7: Further data on primary neurons	13
Figure S8: Further data NK cells	14
Figure S9: Further data on tissue sections	15
Figure S10: PCA of MCF10A panel using subset of lectins	16
Figure S11: PCA on full NN distance distribution for MCF10A panel	17
Figure S12: Reconstruction from live lectin staining using WGA	18

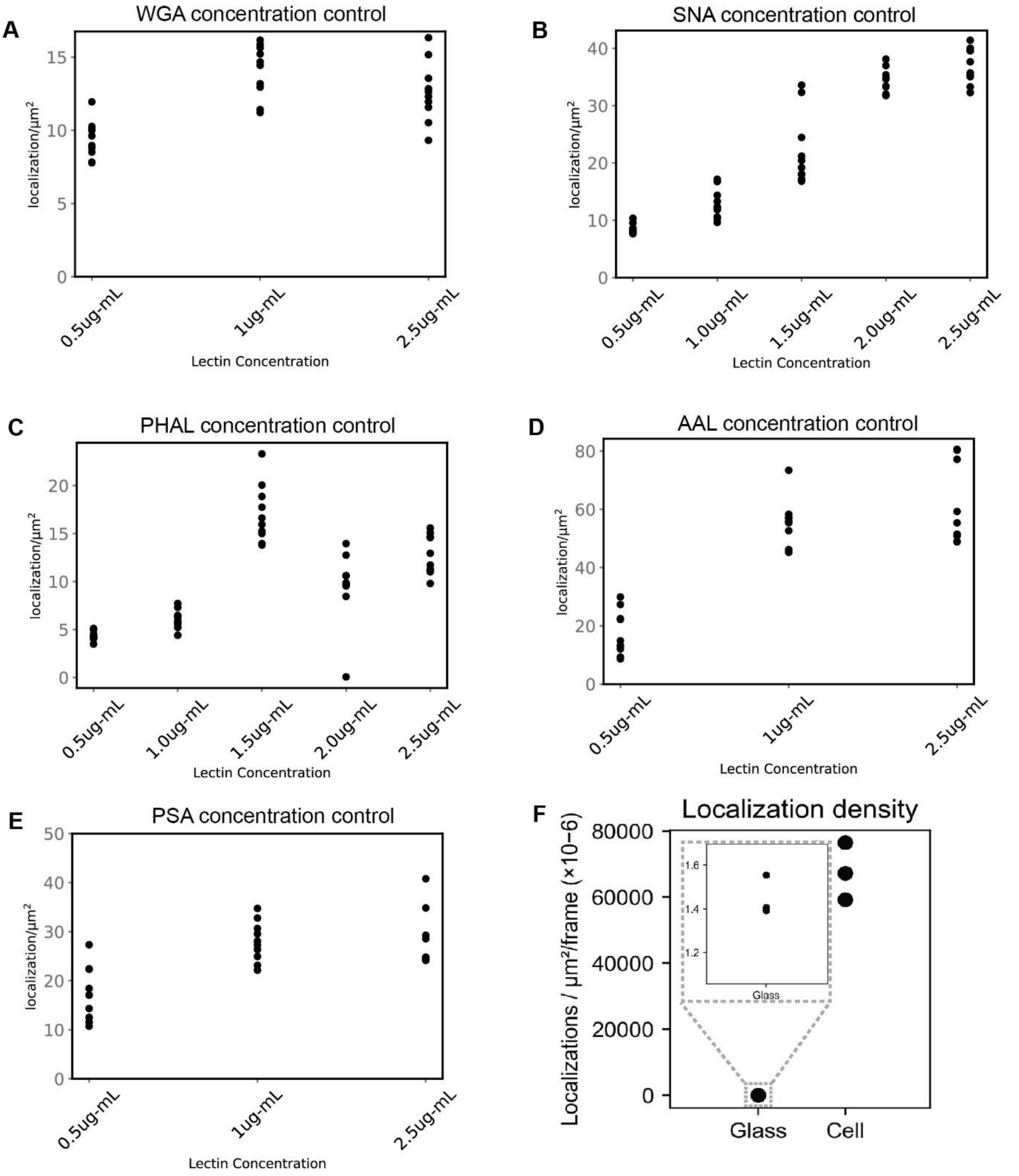

Figure S1: Control experiments for labeling optimization. A-E) Determination of lectin concentration for labeling (see plot titles for lectin type). F) Negative control for lectin affinity to bare glass without cultured cells compared to the cell surface. Note that in A)-E), localization densities are given per square micron for a full stack of 1,000 frames, whereas in F), localization densities are given per square microns per single frame.

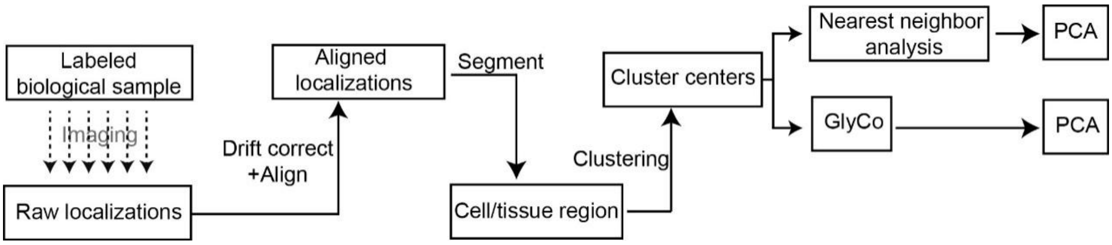

Figure S2: Experimental flow of Glycan Atlassing.

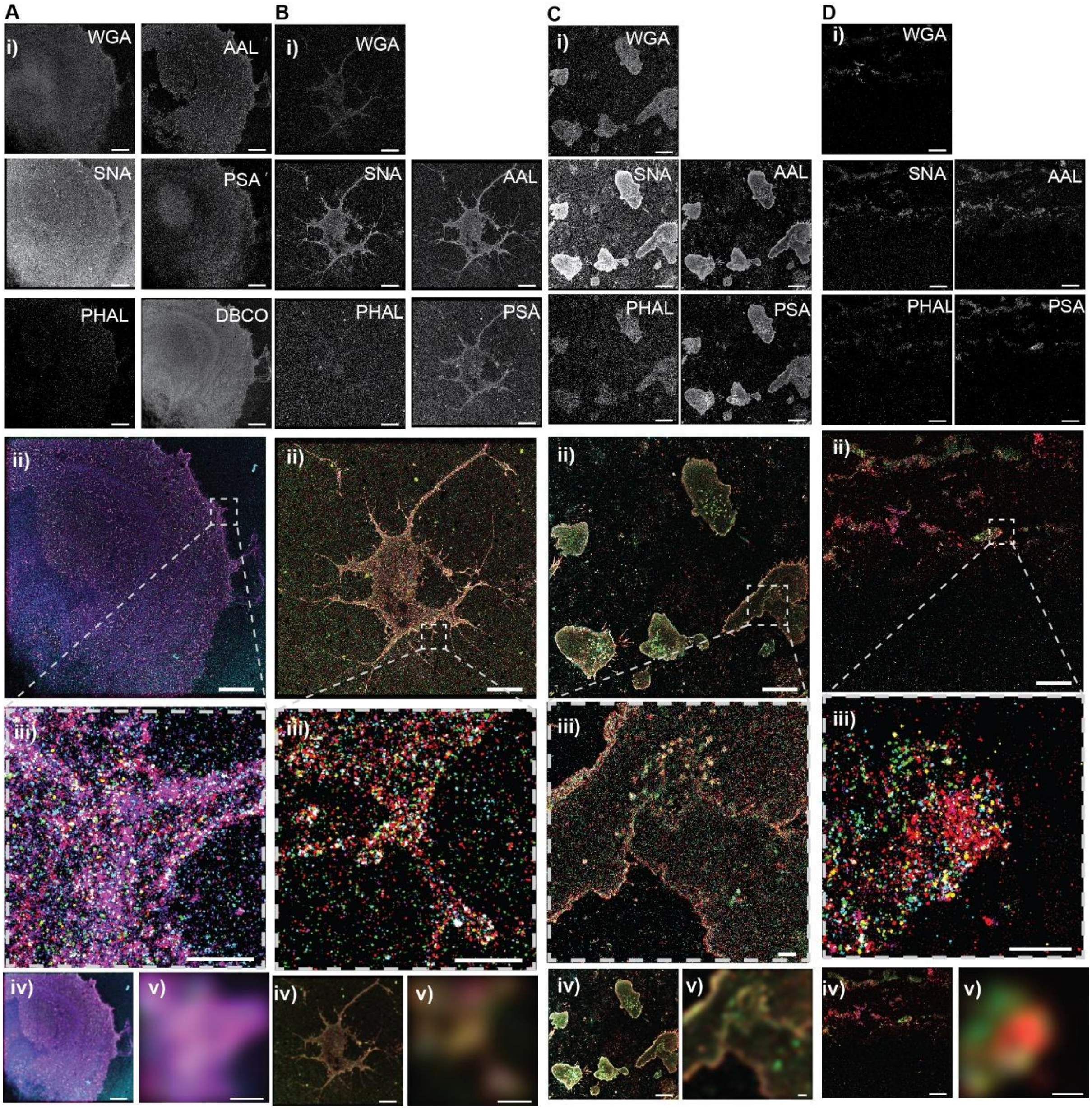

Figure S3: Additional reconstructions across sample types. A-D) Individual channels depicted in grayscale (i), merged channels (ii) using the following color code: WGA – magenta, SNA – cyan, PHA-L – yellow, AAL – red, PSA – green, DBCO – purple. iii) Zoom-in showing intricate details resolved. iv) Diffraction-limited representation of the whole field of view. v) Diffraction-limited zoom-in corresponding to (iii). A) MCF10AT, B) Primary neuron, C) Immune cells, D) Tissue. Scale bars: 10 µm for full field of views and 1 µm for zoom-ins.

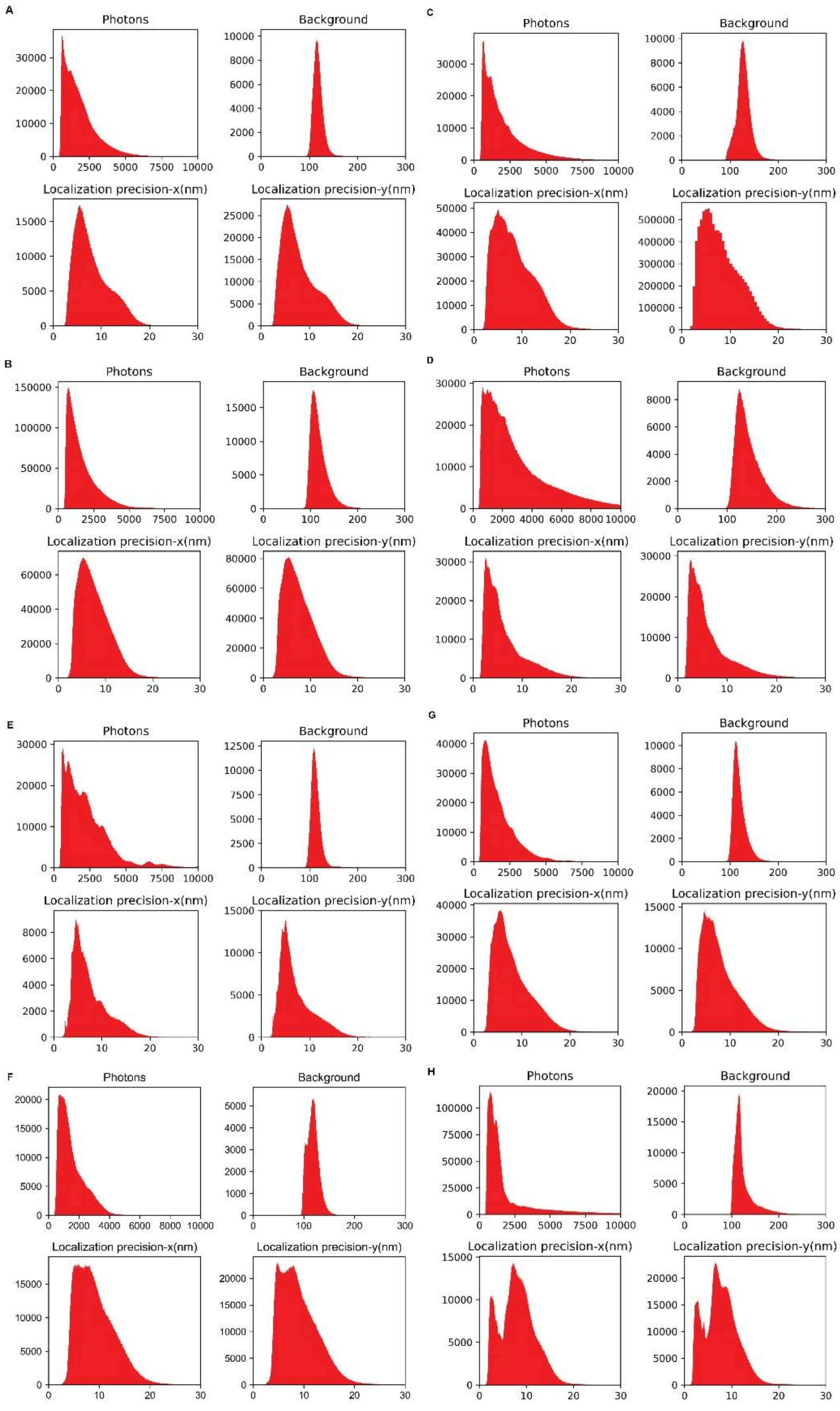

Figure S4: Quality metrics. Signal photon count, background, localization precision in x and y of a representative channel (AAL) for **A)** MCF10A **B)** MCF10A+TGFβ **C)** MCF10AT **D)** MCF10AT+TGFβ **E)** Neurons **F)** Tissue sections **G)** Stimulated NK cells **H)** Non stimulated NK cells **I)** Non stimulated CD4+ cells **J)** Stimulated neutrophils **K)** Non stimulated neutrophils. **L)** Localization precision across channels and across the whole dataset used in the study (see plot titles). Localization precision are given as NeNA precisions.

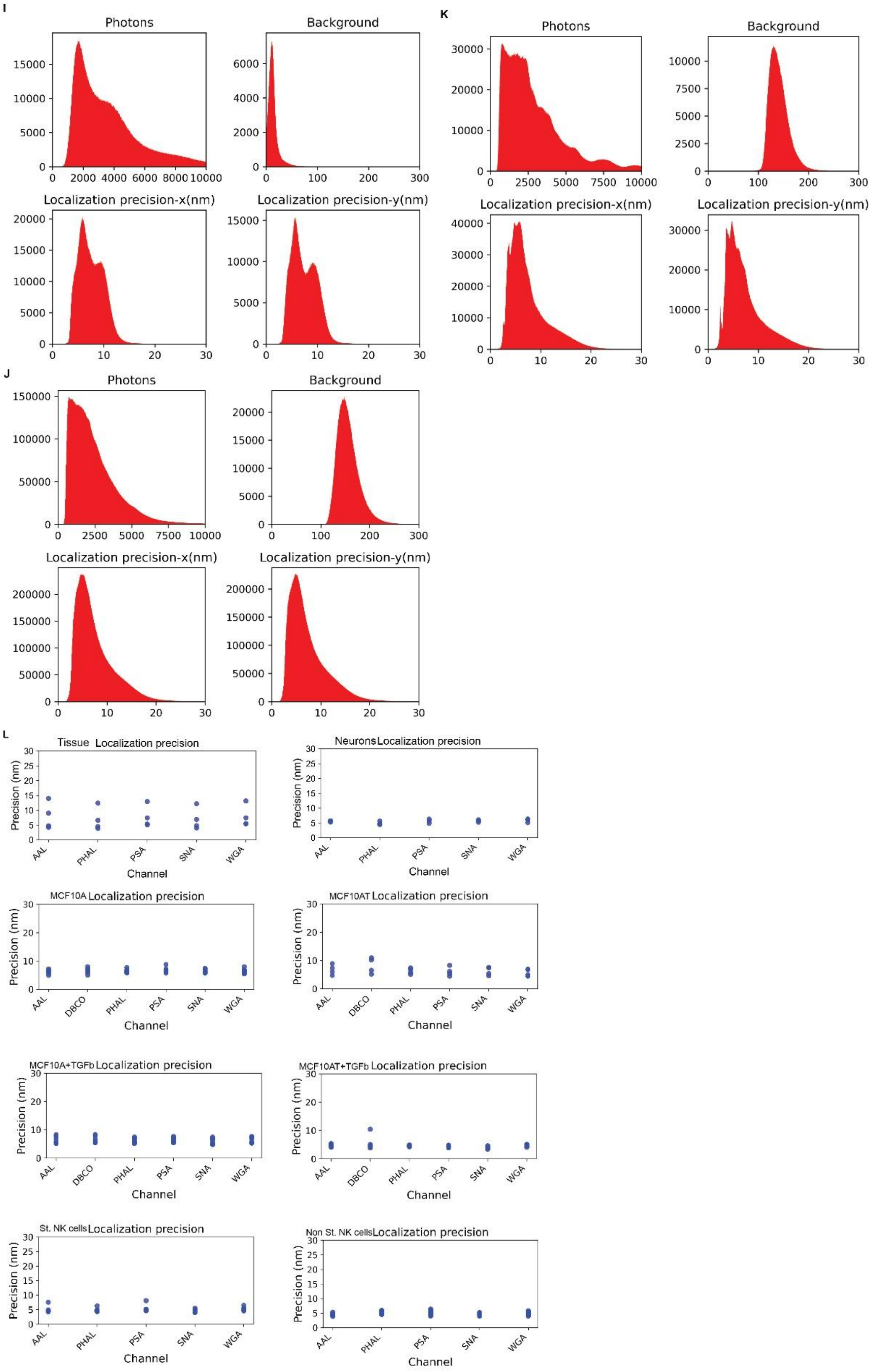

Figure S4: Quality metrics continued.

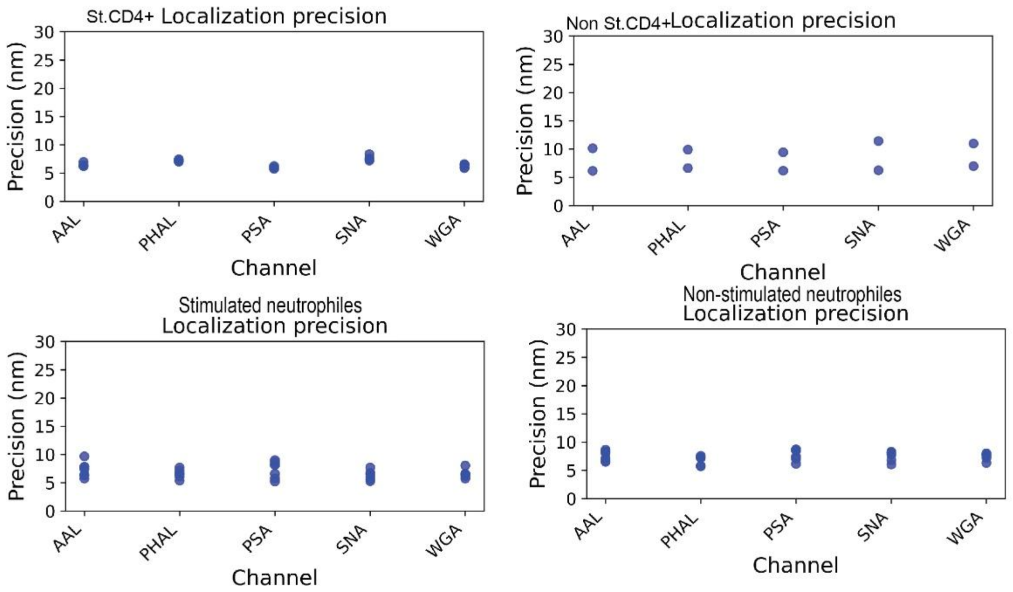

Figure S4: Quality metrics continued.

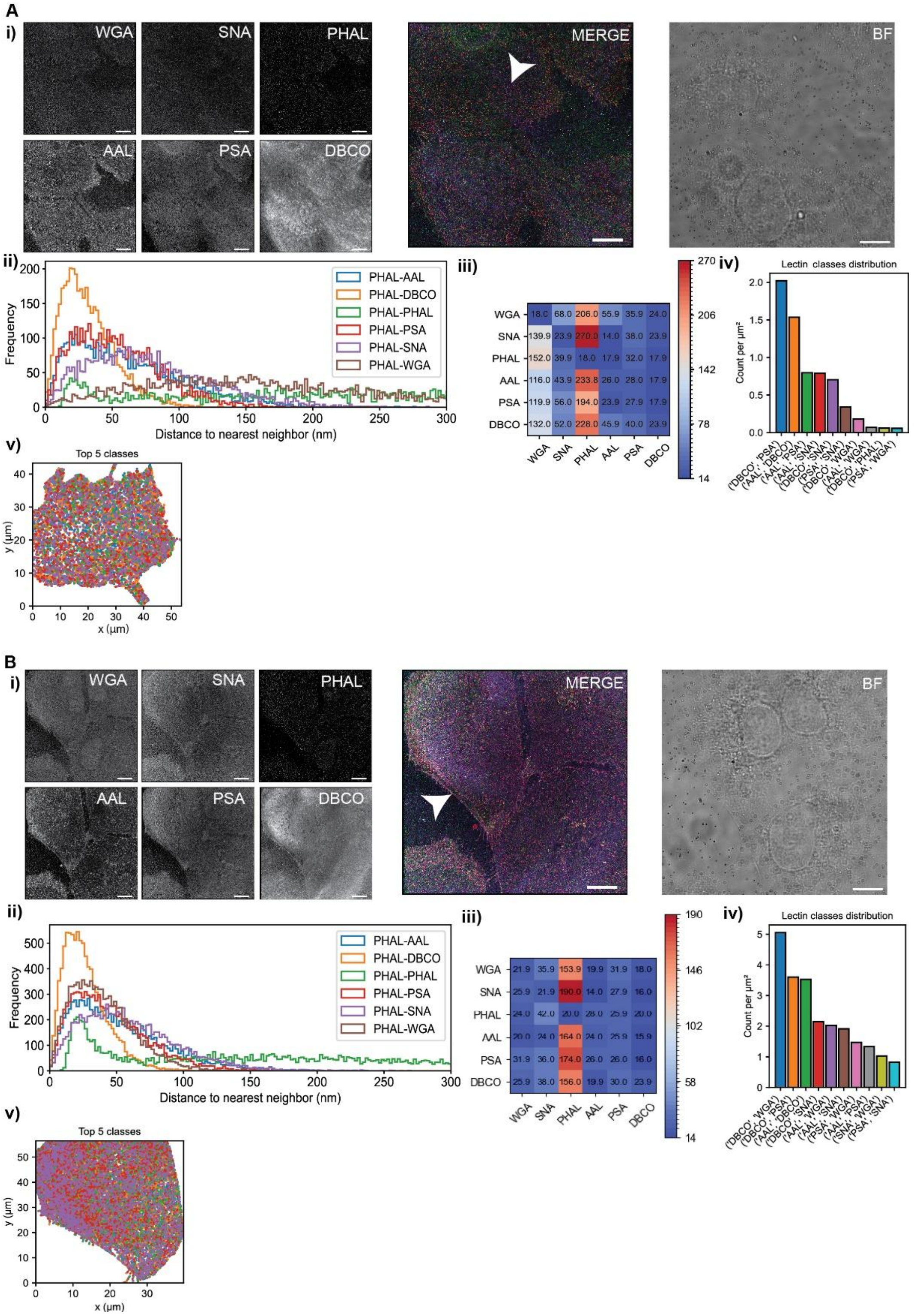

Figure S5: Further data on MCF10A panel. A) MCF10A; **B), C)** MCF10A+TGFβ **D), E)** MCF10AT **F), G)** MCF10AT+TGFβ.

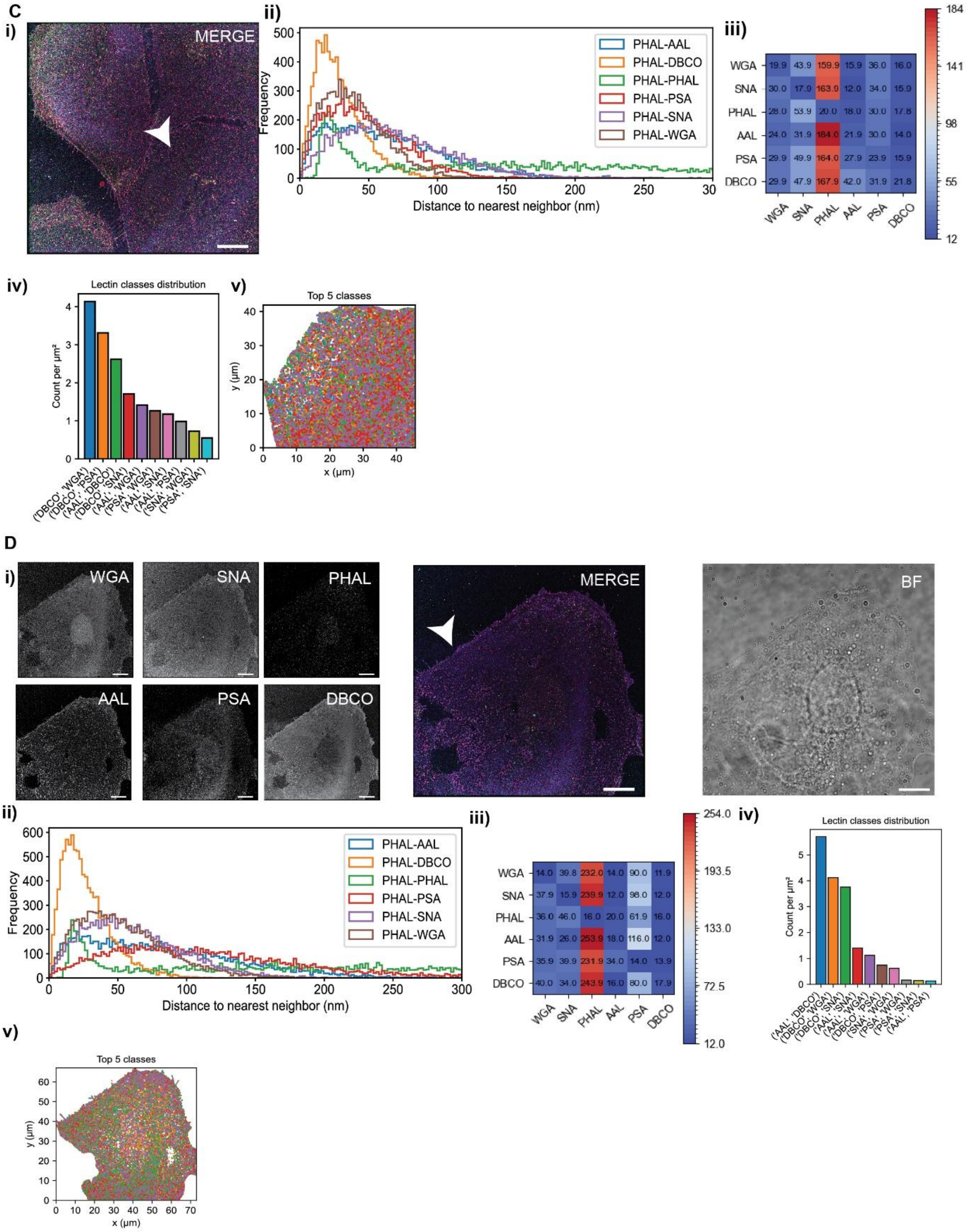

Figure S5: Further data on MCF10A panel continued.

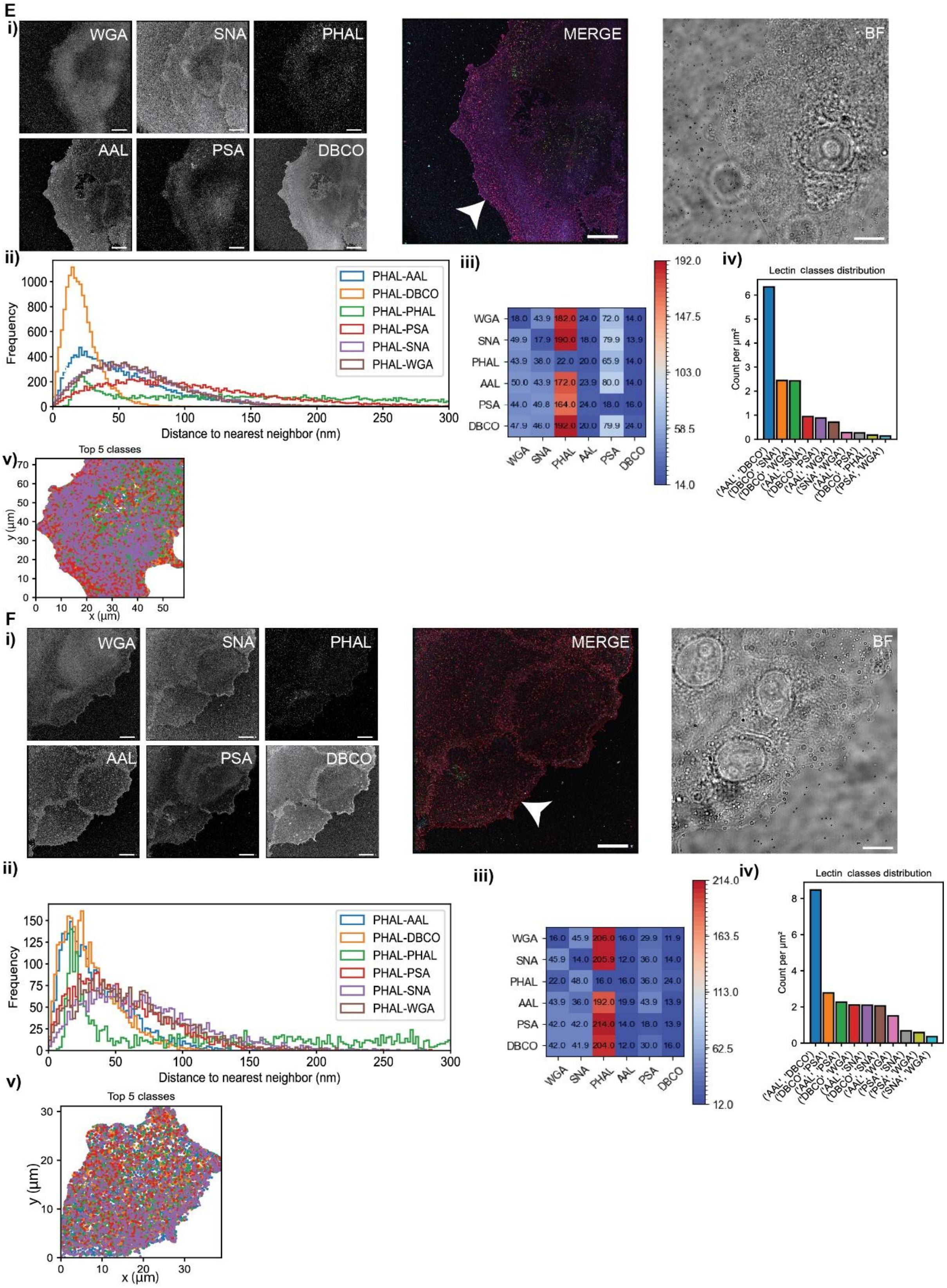

Figure S5: Further data on MCF10A panel continued.

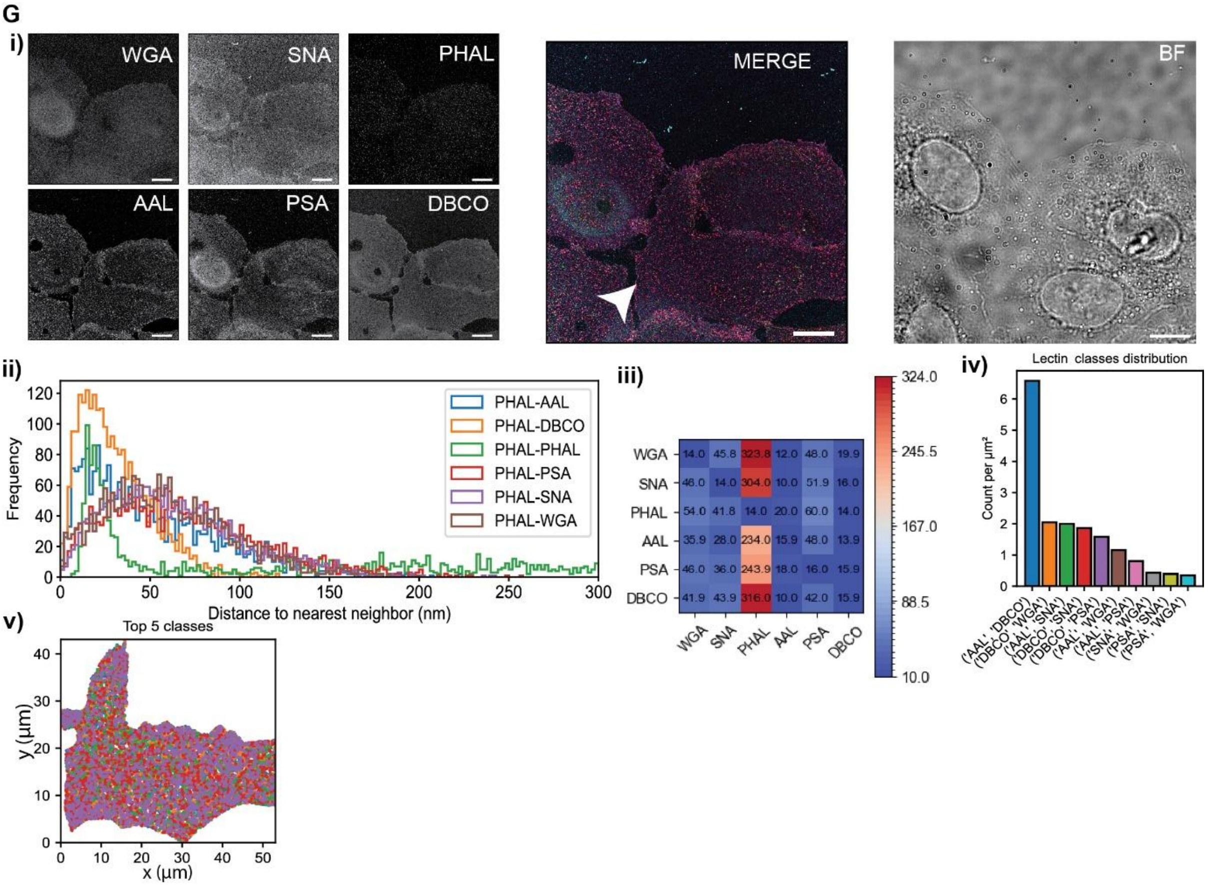

Figure S5: Further data on MCF10A panel continued.

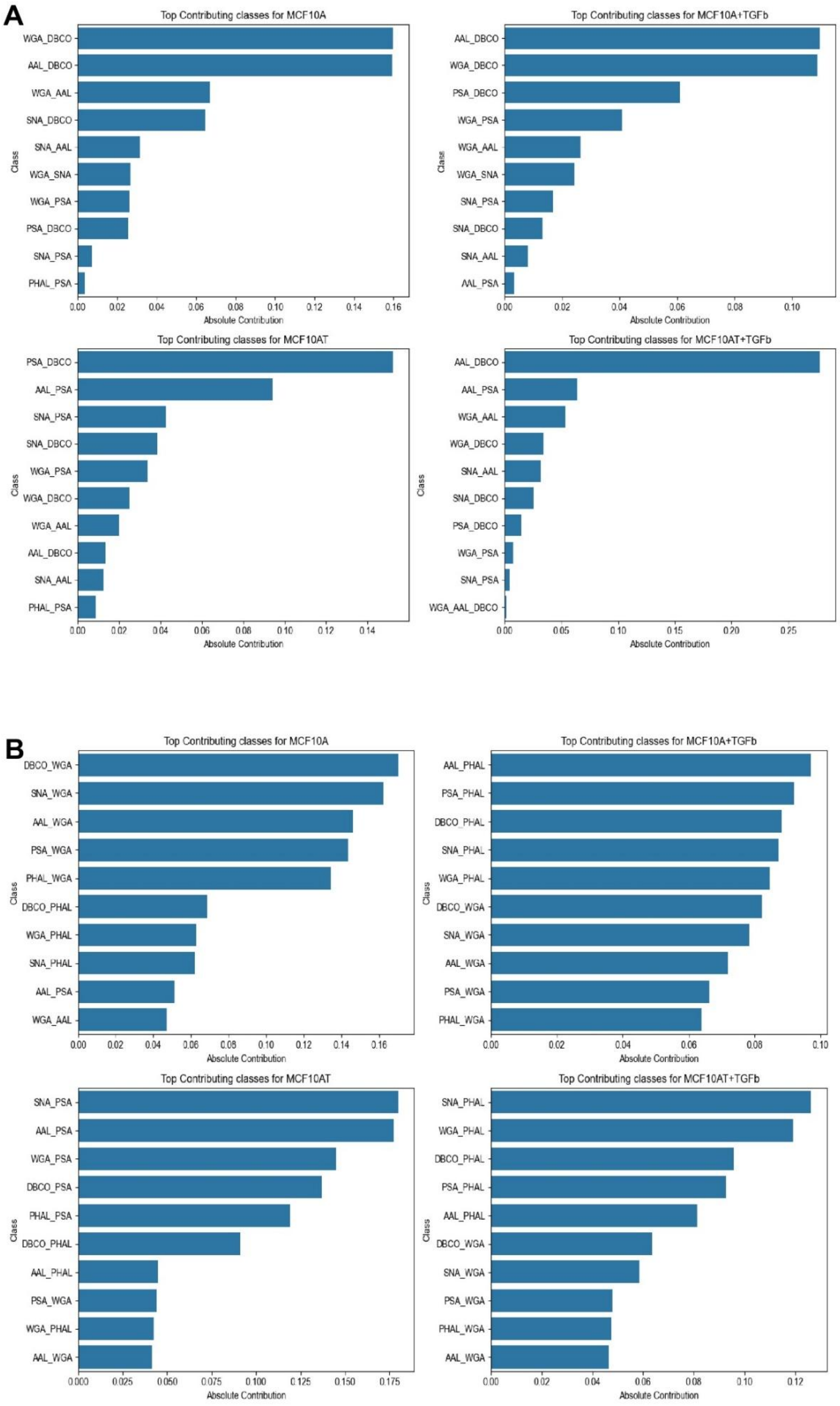

Figure S6: PCA loadings. A) Loadings for GlyCo; B) Loadings for NN distance analysis. In both cases, multiple dimensions show significant contributions.

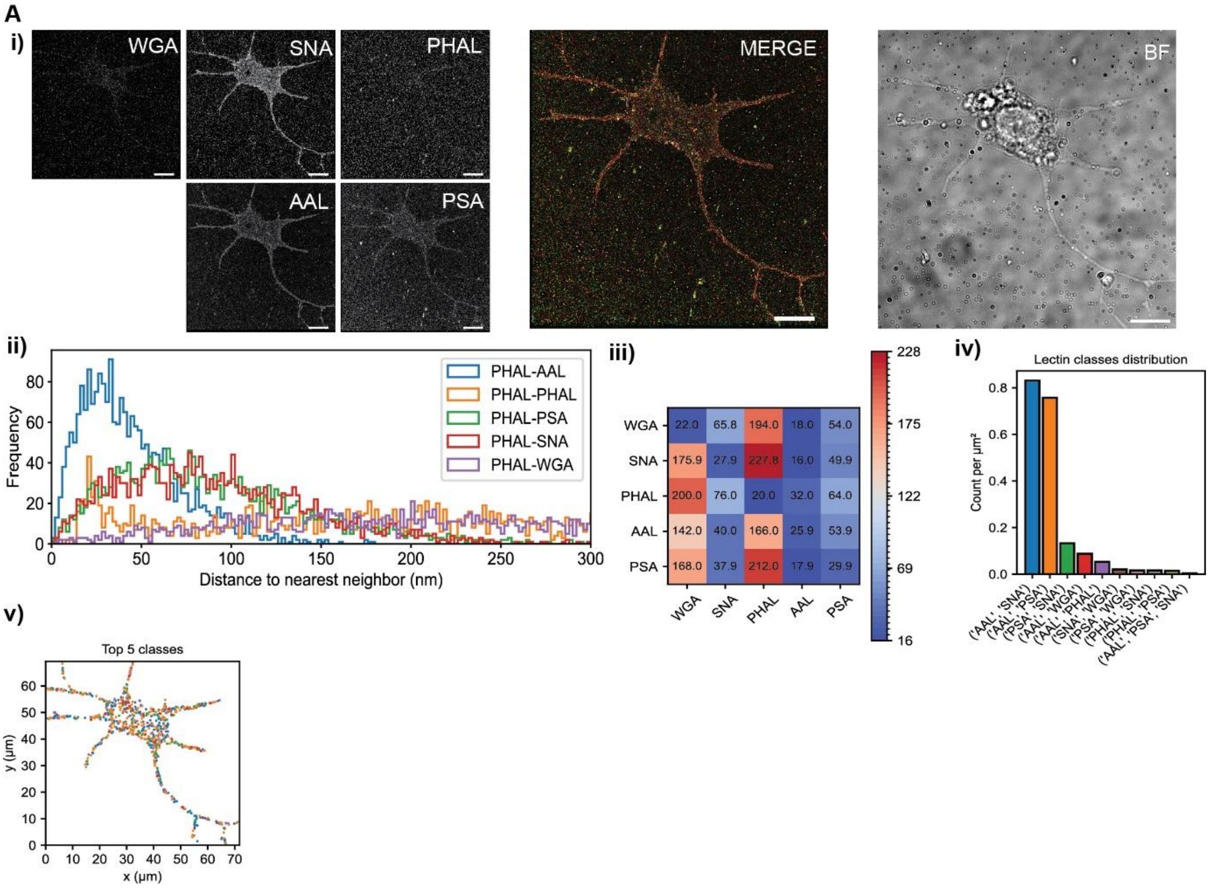

Figure S7: Further data on primary neurons.

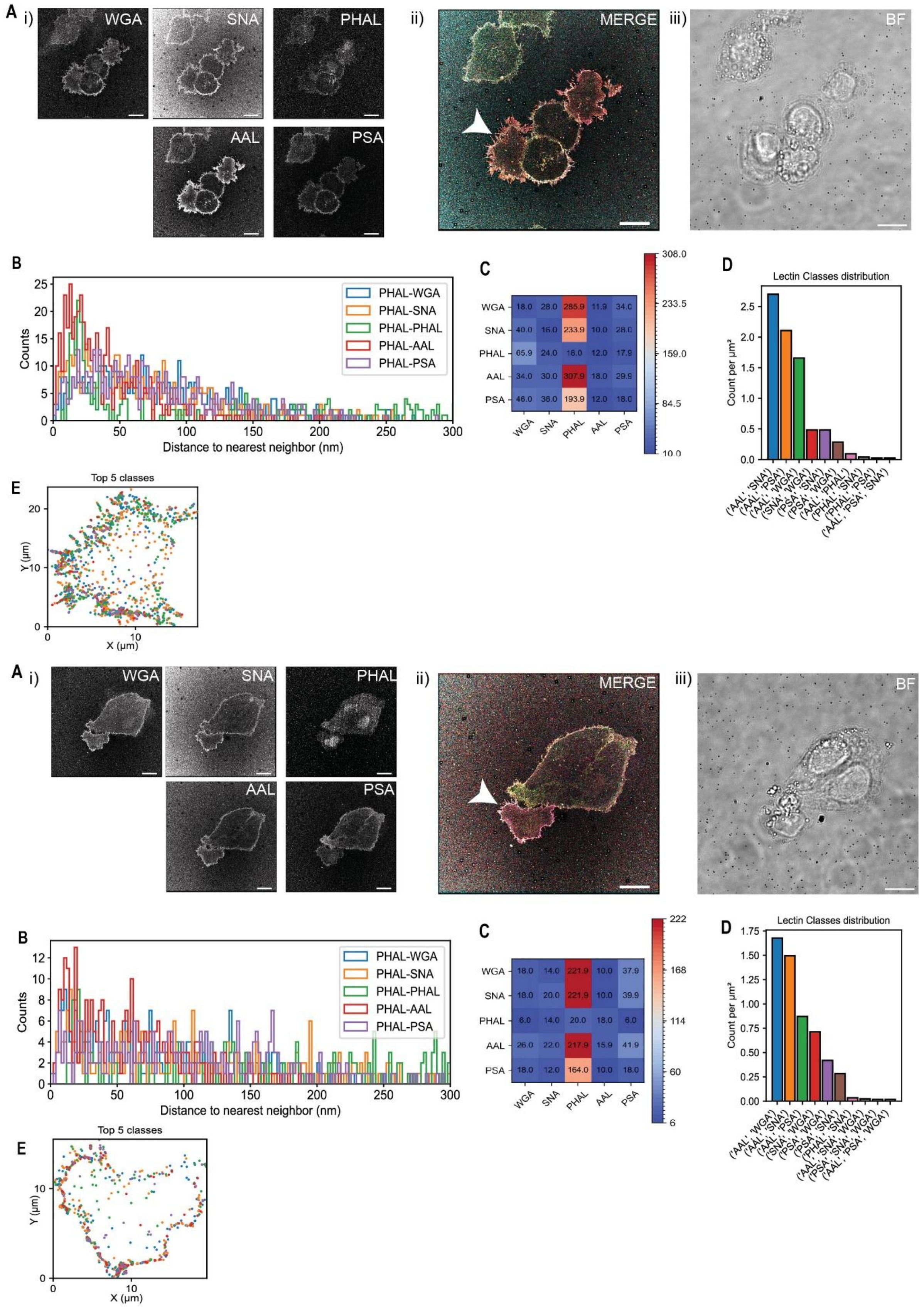

Figure S8: Further data on NK cells. A), B) Two representative fields of view, showing NK cells in co-culture with A549 cells.

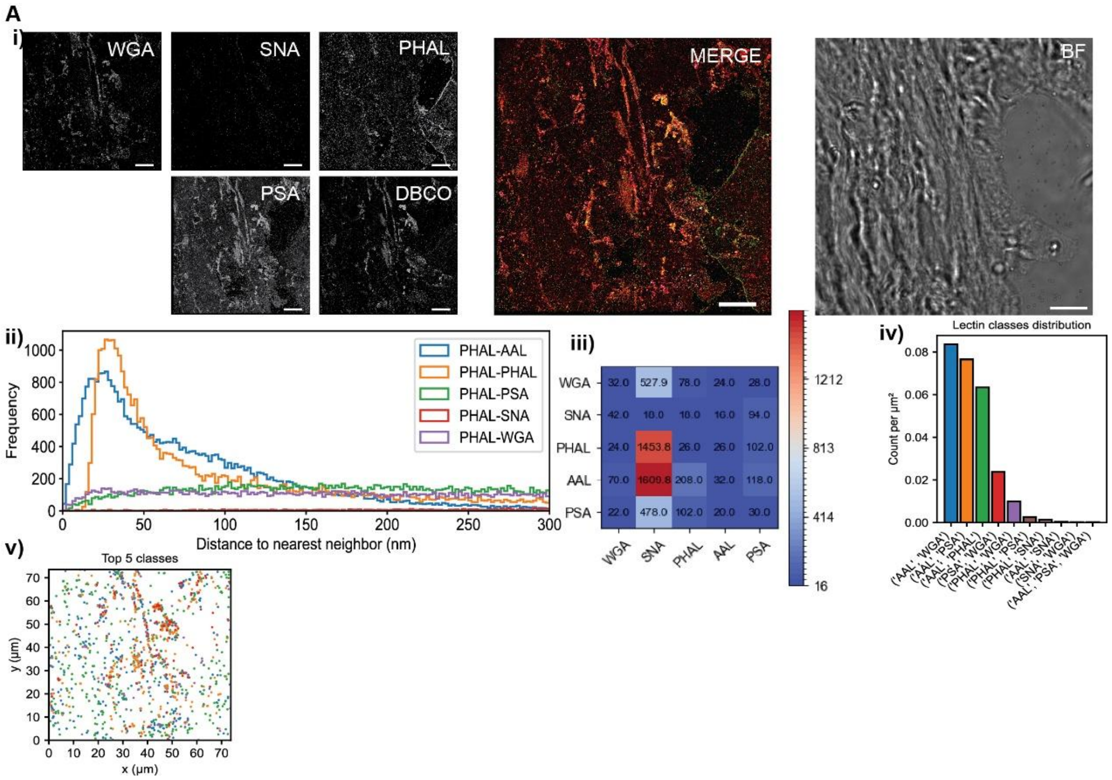

Figure S9: Further data on tissue sections.

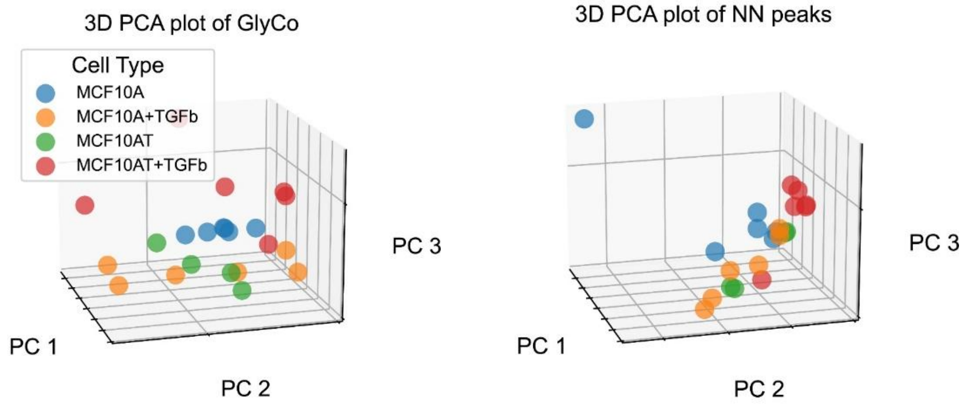

Figure S10: PCA of MCF10A panel using a subset of all lectins. PCA was performed on the dataset using classes from WGA, SNA and AAL only. Left: GlyCo; right: NN distance analysis. In both cases, the separation of conditions becomes significantly worse.

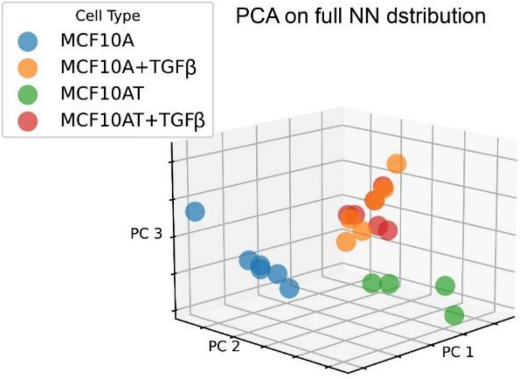

Figure S11: PCA on full NN distance distribution for MCF10A panel. Instead of the peak values of the NN distance histograms, the full distribution was used as input.

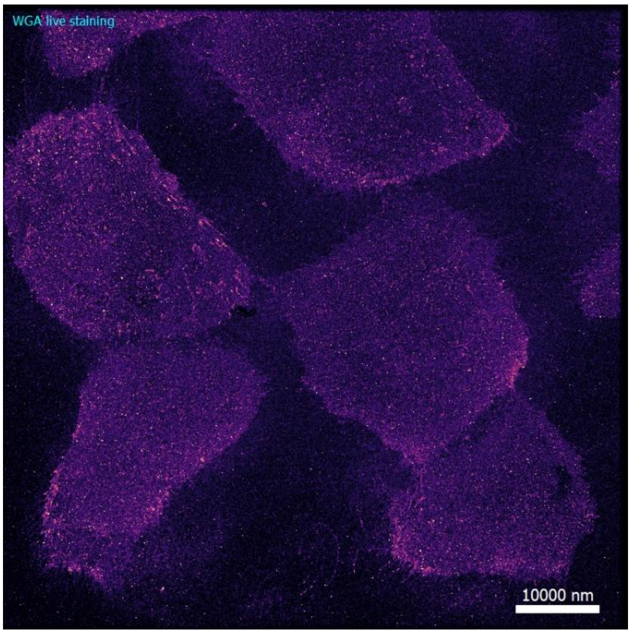

Figure S12: Reconstruction result for live-cell staining followed by fixation. Results for staining first, followed by fixation is shown. All other parameters are identical to the protocol used.
